# Design and Performance Analysis of an SPR Sensor for Milk Adulteration Monitoring

**DOI:** 10.3390/bios16090494

**Published:** 2026-09-04

**Authors:** John Germán Vera Luzuriaga, Marco Guevara, Diana Coello-Fiallos, Cristian Vacacela Gomez

**Affiliations:** 1 Departamento de Ciencias de la Energía y Mecánica, Universidad de las Fuerzas Armadas ESPE, Latacunga 050150, Ecuador; 2 Facultad de Mecánica, Escuela Superior Politécnica de Chimborazo (ESPOCH), Riobamba 060155, Ecuador; 3 Carrera de Física, Grupo GIMA, Facultad de Ciencias, Escuela Superior Politécnica de Chimborazo (ESPOCH), Riobamba 060155, Ecuador; 4 Universidad Ecotec, Km. 13.5 Samborondón, Samborondón 092302, Ecuador

**Keywords:** surface plasmon resonance, hydrogen peroxide, milk adulteration, borosilicate prism, Kretschmann configuration, transfer matrix method, numerical simulation

## Abstract

Hydrogen peroxide (H_2_O_2_) may be illegally added to milk to delay visible spoilage, producing concentration-dependent changes in its refractive index. This study numerically evaluates the optical response of a B-sil/Al/Al_2_O_3_/WS_2_ surface plasmon resonance (SPR) configuration to these refractive-index variations. The angular SPR response was calculated at λ = 633 nm using the transfer matrix method under TM-polarized illumination. The B-sil/Al/Al_2_O_3_/WS_2_ architecture was established through sequential evaluation of prism material, Al thickness, Al_2_O_3_ thickness, and 2D interfacial material, followed by assessment of its response to H_2_O_2_-associated refractive-index changes in milk. The final structure consisted of 70 nm Al, 25 nm Al_2_O_3_, and 0.80 nm WS_2_. Across the simulated H_2_O_2_ conditions, the resonance angle shifted from 85.65° to 86.11°, while the angular sensitivity ranged from 383.82 to 406.45°/RIU. The best balance among the evaluated metrics was obtained for H_2_O_2_-C2, with a sensitivity of 406.45°/RIU, QF of 4.65 RIU^−1^, FoM of 436.48 RIU^−1^, LoD of 1.23 × 10^−5^, and CSF of 436.56. The WS_2_-containing interface produced calculated angular shifts for small refractive-index variations in the milk sensing medium. Comparison with reported SPR systems for milk-related sensing showed a comparable angular sensitivity range, while QF and detection accuracy were limited by the broad resonance profile. These results characterize the theoretical optical response of the B-sil/Al/Al_2_O_3_/WS_2_ multilayer under H_2_O_2_-associated refractive-index changes but do not establish chemical selectivity toward H_2_O_2_. Experimental implementation would require an appropriate selective filtering or recognition strategy, together with validation of matrix effects and practical sensing performance.

## 1. Introduction

Milk is a widely consumed food that provides proteins, lipids, lactose, minerals, and vitamins essential for human nutrition [1,2,3]. Its nutrient-rich composition also makes it susceptible to microbial growth during collection, transport, and storage [4,5]. Poor refrigeration or handling can accelerate spoilage and motivate the illegal addition of preservatives such as hydrogen peroxide (H_2_O_2_), whose oxidizing and antimicrobial activity can delay visible deterioration while masking deficiencies in hygienic processing [6,7,8,9]. Residual H_2_O_2_ may alter milk constituents and compromise product quality, making its detection relevant to food safety and adulteration control [10,11,12,13,14,15].

Several analytical approaches have been applied to preservative and adulterant detection in milk, including colorimetric, electrochemical, chromatographic, spectrophotometric, and optical methods [16,17,18,19,20,21,22,23,24,25,26]. Some of these techniques require reagents, sample pretreatment, trained personnel, or laboratory infrastructure [27,28]. Refractive-index-based optical sensing provides a label-free approach for liquid food matrices, and previous studies have shown that the refractive index of milk varies with the concentration of preservatives such as H_2_O_2_, formaldehyde, and sodium carbonate [18,25,29,30,31].

Surface plasmon resonance (SPR) detects refractive-index changes at a metal–dielectric interface [32,33,34]. In the Kretschmann configuration, p-polarized light is coupled through a prism to a thin metallic film, producing a reflectance minimum at the resonance angle [35,36]. Variations in the refractive index of the sensing medium shift this angle and provide the basis for analyte monitoring [33,37,38,39]. SPR performance is commonly characterized using angular sensitivity, full width at half maximum (FWHM), detection accuracy, and figure of merit, including in theoretical studies of preservative detection in milk [31,40,41].

Al/Al_2_O_3_ interfaces have also been investigated in plasmonic sensing systems because of their optical properties and compatibility with thin-film configurations [42,43]. Optical approaches for H_2_O_2_ detection have been reported using different plasmonic transduction mechanisms, including SPR imaging and surface-enhanced Raman scattering [44,45]. These studies show that aluminum-based plasmonic sensing and optical H_2_O_2_ detection are established research directions. The studies considered here, however, employ different material combinations, interrogation mechanisms, or sensing matrices from the B-sil/Al/Al_2_O_3_/WS_2_ angular SPR configuration examined in the present work.

The present study therefore focuses on the numerical evaluation of a planar B-sil/Al/Al_2_O_3_/WS_2_ multilayer structure in the Kretschmann configuration for monitoring refractive-index changes associated with H_2_O_2_ adulteration in milk. In this architecture, the borosilicate prism acts as the optical coupling element that enables phase matching between the incident wave and the surface plasmon mode [46]. Its optical transparency at the operating wavelength, mechanical stability, and compatibility with thin-film deposition support its use as the substrate for the multilayer configuration [47,48]. A thin aluminum film serves as the plasmonic layer [49], while Al_2_O_3_ is introduced as a dielectric coating and potential protective barrier against oxidation-related degradation of Al [50,51]. WS_2_ is incorporated as a two-dimensional interfacial layer to modify the evanescent-field distribution near the sensing region and enhance the interaction between the optical field and the sensing medium [52,53]. The sensing medium is represented by milk containing H_2_O_2_, where concentration-dependent refractive-index variations are monitored through the corresponding displacement of the SPR angle [7,31].

The multilayer architecture is systematically evaluated through sequential analysis of prism material, Al thickness, Al_2_O_3_ thickness, and 2D interfacial material, followed by assessment of its angular SPR response over the modeled H_2_O_2_-associated refractive-index range in milk. This configuration differs from previously reported approaches in the combination of multilayer materials, angular interrogation scheme, and milk-based sensing matrix considered. The final structure is evaluated at λ = 633 nm using resonance-angle shift, angular sensitivity, FWHM, detection accuracy, figure of merit, and limit of detection. The resulting system is examined as a theoretical optical platform for H_2_O_2_-associated refractive-index monitoring in milk, with experimental validation required to establish its practical sensing performance.

## 2. Materials and Methods

### 2.1. Theoretical Modelling and SPR Sensor Configuration

**Scheme 1:**
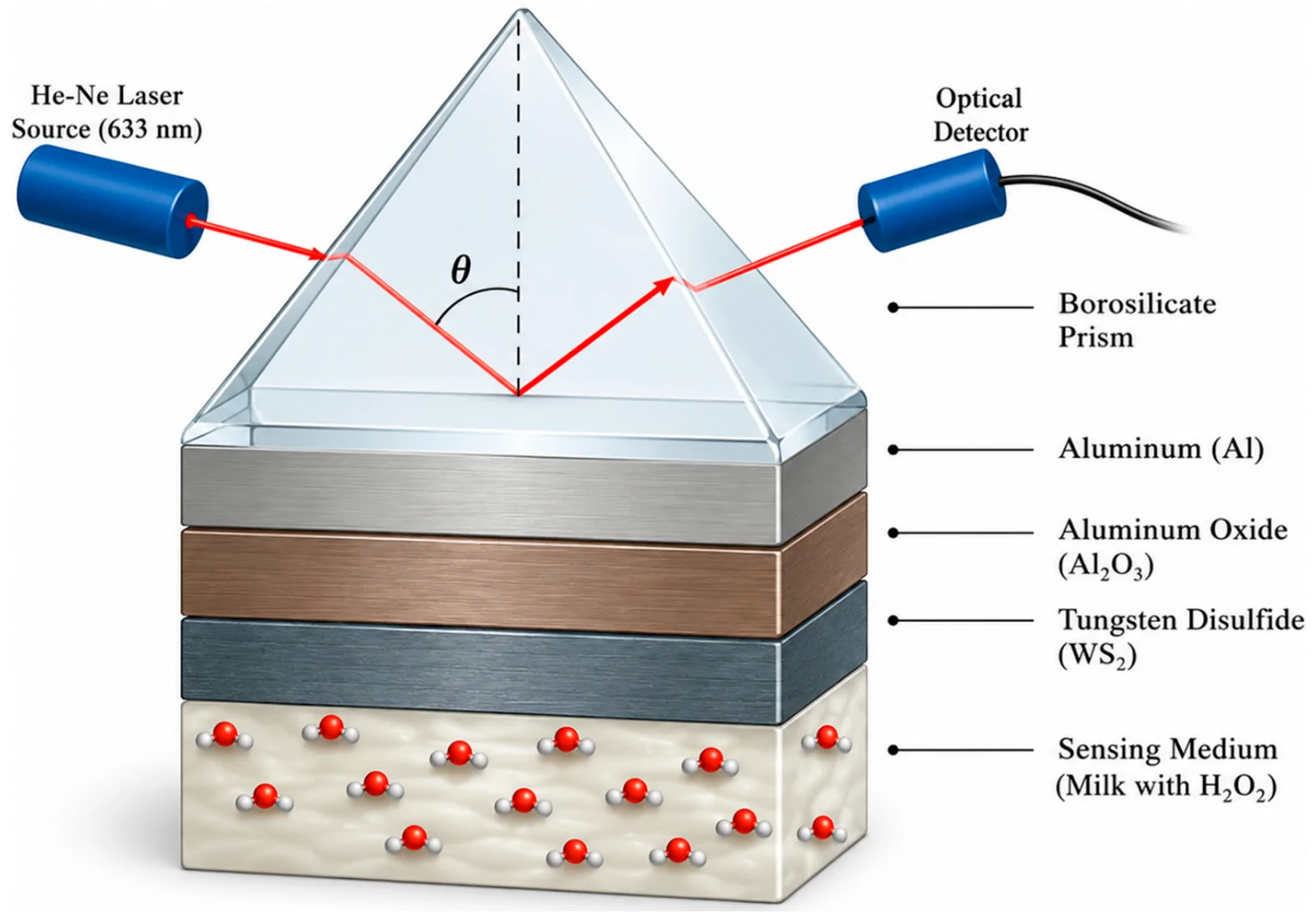
Proposed borosilicate prism-based SPR configuration for modeling H_2_O_2_-associated refractive-index changes in milk.

The proposed multilayer configuration was modeled using the Kretschmann geometry for angular surface plasmon resonance interrogation. Scheme 1 shows the multilayer architecture, consisting of a borosilicate prism, an aluminum layer, an aluminum oxide coating, a tungsten disulfide interfacial layer, and the sensing medium formed by milk containing H_2_O_2_. A He–Ne laser source operating at λ = 633 nm was considered as the incident optical beam. The light was assumed to be transverse magnetic polarized, because only this polarization can couple efficiently with surface plasmon modes at the metal–dielectric interface. The incidence angle, θ, was varied to identify the resonance condition from the angular reflectance minimum.

Table 1 summarizes the optical constants and physical thicknesses used for the final B-sil/Al/Al_2_O_3_/WS_2_ configuration. The refractive indices correspond to the operating wavelength of 633 nm. The final metal and dielectric thicknesses were 70 nm for Al, 25 nm for Al_2_O_3_, and 0.80 nm for WS_2_. These values were not imposed a priori; they were retained after the sequential evaluation of prism material, Al thickness, Al_2_O_3_ thickness, and 2D interfacial material presented in the subsequent Results subsections. The corresponding screening and optimization trends are discussed in Section 3.1, Section 3.2, Section 3.3, Section 3.4, Section 3.5 and Section 3.6.

The metallic layer was selected as aluminum because it supports plasmonic excitation at visible wavelengths and provides a low-cost alternative to noble metals. Since aluminum is prone to surface oxidation, an Al_2_O_3_ coating was introduced above the Al film. In the model, this layer functions as both a dielectric spacer and a protective interface that can reduce direct degradation of the aluminum surface. The WS_2_ layer was placed at the outer interface of the multilayer stack. As a two-dimensional transition-metal dichalcogenide, WS_2_ modifies the evanescent-field distribution near the sensing region and increases the interaction between the optical field and the analyte-containing medium.

The sensing medium was modeled as milk adulterated with hydrogen peroxide. Changes in H_2_O_2_ concentration were represented through refractive-index variations relative to the baseline milk value. When the refractive index of the sensing medium changes, the phase-matching condition is altered and the SPR angle shifts. This resonance-angle displacement is the main output used to evaluate the optical response of the B-sil/Al/Al_2_O_3_/WS_2_ configuration. The structure shown in Scheme 1 is a theoretical configuration intended for numerical analysis. Experimental implementation would require controlled deposition of the Al, Al_2_O_3_, and WS_2_ layers, calibration with reference liquids and milk samples containing known H_2_O_2_ concentrations, and assessment of selectivity under realistic matrix conditions.

### 2.2. Numerical Modeling

The optical response of the proposed SPR configuration was evaluated using the transfer matrix method (TMM). The multilayer structure was modeled as a B-sil prism/Al/Al_2_O_3_/WS_2_/sensing-medium stack, where each layer was defined by its refractive index, dielectric constant, and physical thickness. The TMM calculations assume planar, homogeneous, isotropic, and optically uniform layers with abrupt interfaces and fixed optical constants at the operating wavelength. Surface roughness, thickness nonuniformity, interfacial defects, and material degradation are not included in the present model.

At each interface, the tangential components of the electric and magnetic fields were assumed to be continuous. Accordingly, the field components at the first and final interfaces of the multilayer system were related through the global transfer matrix *M* as

(1) $$\left[\begin{array}{c} E_{1}\\ H_{1} \end{array}\right]=M_{ij}\left[\begin{array}{c} E_{N-1}\\ H_{N-1} \end{array}\right]$$

where $E_{1}$ and $H_{1}$ are the tangential electric and magnetic field components at the first interface, while $E_{N-1}$ and $H_{N-1}$ correspond to those at the final interface. The global transfer matrix in Equation (1) was obtained from the product of the characteristic matrices of the intermediate layers, as given in Equation (2):

(2) $$M_{ij}={\left[\prod_{k=2}^{N-1}M_{k}\right]}_{ij}=\left[\begin{array}{cc} M_{11} & M_{12}\\ M_{21} & M_{22} \end{array}\right]$$

For TM-polarized illumination, the characteristic matrix of the kth layer was defined in Equation (3):

(3) $$M_{k}=\left[\begin{array}{cc} \cos\beta_{k} & (-i\;\sin\beta_{k})/q_{k}\\ -i\;q_{k}\sin\beta_{k} & \cos\beta_{k} \end{array}\right]$$

where $q_{k}$ is the optical admittance and $\beta_{k}$ is the phase thickness of the kth layer.

The optical admittance $q_{k}$ and the phase thickness $\beta_{k}$ were calculated using Equations (4) and (5), respectively:

(4) $$\beta_{k}=\frac{2\pi d_{k}}{\lambda_{0}}\sqrt{\varepsilon_{k}-n_{1}^{2}\sin^{2}\theta }$$

(5) $$q_{k}=\frac{\sqrt{\varepsilon_{k}-n_{1}^{2}\sin^{2}\theta }}{\varepsilon_{k}}$$

where $\varepsilon_{k}$ is the dielectric constant, d_k_ is the layer thickness, n_1_ is the refractive index of the incident medium, θ is the incident angle, and λ is the operating wavelength.

The TM reflection coefficient was calculated from the elements of the global matrix using Equation (6):

(6) $$R={\left|\frac{\left(M_{11}+M_{12}\;q_{N}\right)q_{1}-\left(M_{21}+M_{22}\;q_{N}\right)}{\left(M_{11}+M_{12}\;q_{N}\right)q_{1}+\left(M_{21}+M_{22}\;q_{N}\right)}\right|}^{2}$$

Using the TM reflection coefficient obtained from Equation (6), the angular reflectance was calculated in Equation (7):

(7) $$R_{p}={\left|r_{p}\right|}^{2}$$

where $R_{p}$ is the reflectance for TM-polarized light and $r_{p}$ is the TM reflection coefficient obtained from the TMM formulation. The resonance angle, θ_res_, was defined as the incident angle at which $R_{p}$ reaches its minimum value. Changes in H_2_O_2_ concentration in milk were represented by refractive-index variations in the sensing medium, which produce shifts in θ_res_. All SPR curves were calculated using 5 × 10^4^ angular sampling points to identify the resonance minimum consistently across the evaluated H_2_O_2_ concentration conditions. To assess the numerical implementation independently from the proposed B-sil/Al/Al_2_O_3_/WS_2_ configuration, the TMM model was also compared with previously published experimental SPR data reported in Ref. [38], using the corresponding optical configuration and material parameters described in that study. The comparison between the calculated and experimental reflectance responses is presented in Supplementary Figure S1.

### 2.3. Performance Metrics and Optimization Protocol

The sensor performance was evaluated using the resonance-angle shift, angular refractive-index sensitivity, full width at half maximum, detection accuracy, figure of merit, and limit of detection. The angular refractive-index sensitivity was calculated using Equation (8):

(8) $$S_{RI}=\frac{∆\theta_{res}}{∆n}\;({}^{\circ}{\;RIU}^{-1})$$

where $S_{RI}$ is the angular refractive-index sensitivity, $∆\theta_{res}$ is the resonance-angle shift, and Δn is the refractive-index change of the sensing medium.

The detection accuracy was obtained from the full width at half maximum of the SPR curve using Equation (9):

(9) $$DA=\frac{∆\theta }{FWHM}\;(/{}^{\circ})$$

where DA is the detection accuracy and FWHM is the angular full width at half maximum of the resonance dip.

The figure of merit was calculated using Equation (10):

(10) $$FoM=\frac{S_{RI}(1-R_{min})}{FWHM}\;({\;RIU}^{-1})$$

where $R_{min}$ represents the lowest normalized reflectance value at resonance.

The limit of detection was estimated using Equation (11):

(11) $$LoD=\frac{∆n}{∆\theta }\times 0.005{}^{\circ}\;(RIU)$$

where 0.005° is the minimum resolvable angular shift considered in the numerical analysis. The optimization protocol was based on sequential variation of the multilayer structure. The Al layer was optimized first to obtain a well-defined SPR dip with low minimum reflectance. The Al_2_O_3_ layer was then evaluated as a dielectric and protective coating, followed by the WS_2_ layer as the two-dimensional interfacial material. The final configuration was selected by considering high *S_RI_*, low *FWHM*, high *DA*, high *FoM*, and low *LoD*. The same numerical conditions were applied to all configurations to allow direct comparison.

## 3. Results and Discussion

### 3.1. Prism Selection and Baseline SPR Response

The prism material was evaluated as the first design variable because it controls the optical coupling condition in the Kretschmann configuration. The refractive indices used for the prism-screening stage and the reference media are summarized in Table 2. The tested structures are defined in Table S1. Sys0-BK7 was used as the water-reference structure, while Sys1-BaF_2_, Sys2-CaF_2_, Sys3-B-sil, and Sys4-BK7 correspond to Al-based SPR configurations in contact with the sensing medium. The sensing medium represents the baseline milk sample before the refractive-index variation associated with H_2_O_2_ adulteration.

**Figure 1:**
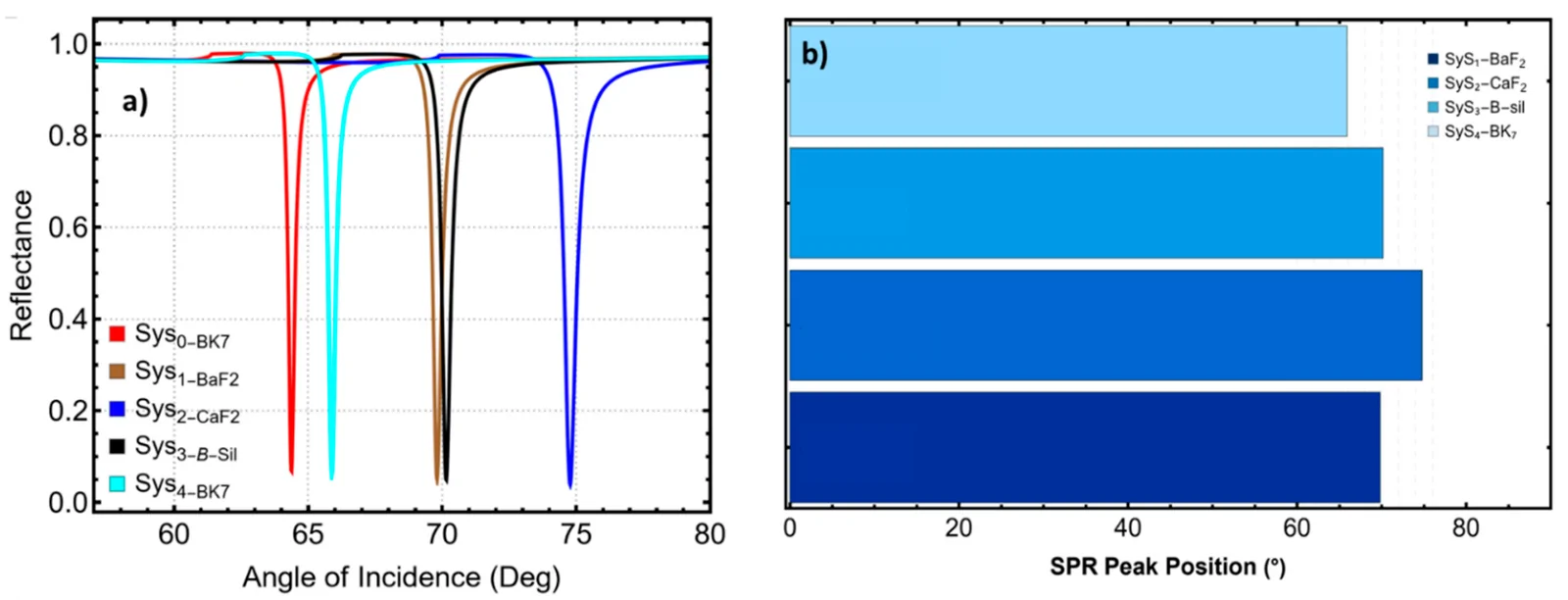
Effect of prism material on the baseline SPR response: (**a**) angular reflectance curves for BK7/Al/H_2_O and prism/Al/sensing-medium configurations and (**b**) SPR peak position obtained for BaF_2_, CaF_2_, B-sil glass, and BK7 substrates.

Figure 1a shows that all prism materials produce a distinguishable SPR dip under angular interrogation at 633 nm. The position of this dip depends on the refractive index of the coupling prism listed in Table 2. The BK7/water reference structure shows the resonance near 64.73°, while replacing water with the sensing medium shifts the resonance to 65.88° for BK7/Al/SM. The other prism materials shift the resonance to higher angles, with values of 69.81° for BaF_2_, 74.77° for CaF_2_, and 70.15° for B-sil glass, as summarized in Table 3. This trend confirms that the prism refractive index modifies the phase-matching condition between the incident optical wave and the surface plasmon mode.

**Figure 2:**
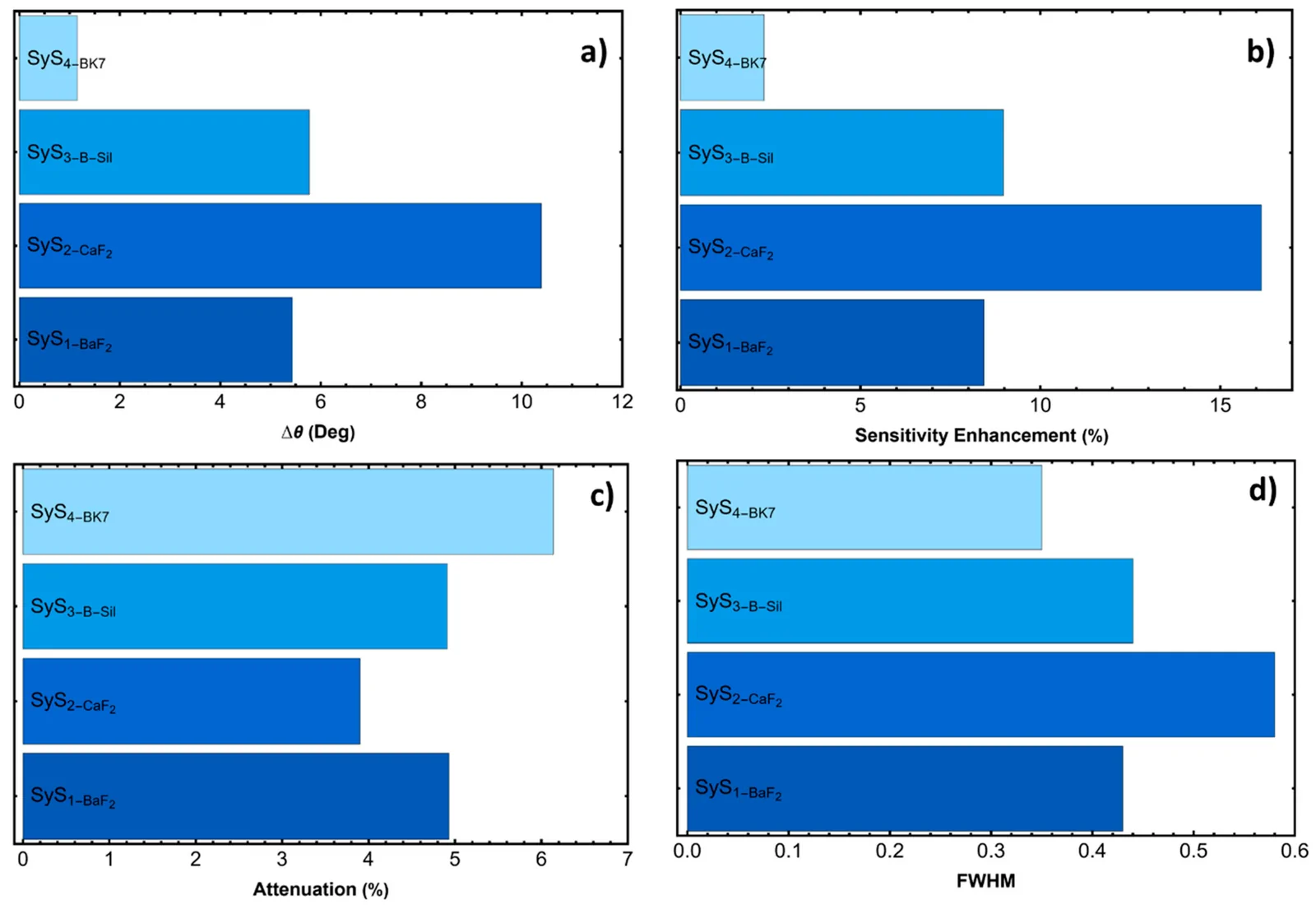
Prism-dependent sensing metrics of the Al-based SPR platform: (**a**) resonance-angle shift, Δθ_SPR_, (**b**) sensitivity enhancement, (**c**) Attenuation, and (**d**) FWHM.

The resonance-angle shift, ΔθSPR, provides a direct measure of the response generated by changing the external medium from water to the milk baseline. As shown in Figure 2a and Table 3, CaF_2_ gives the largest ΔθSPR of 10.39°, followed by B-sil glass with 5.77°, BaF_2_ with 5.43°, and BK7 with 1.15°. The same order is observed for sensitivity enhancement, where CaF_2_ reaches 16.14%, B-sil glass reaches 8.97%, BaF_2_ reaches 8.43%, and BK7 reaches 2.32% (Figure 2b). These results indicate that CaF_2_ gives the strongest angular displacement during the prism-screening stage, while B-sil glass provides an intermediate response that remains clearly separated from the BK7-based sensing case.

The attenuation and resonance width were also considered because a useful SPR configuration requires both a measurable angular shift and a well-defined resonance profile. The attenuation values range from 3.90% for CaF_2_ to 6.14% for BK7, with B-sil glass giving 4.91% (Figure 2c and Table 3). The FWHM values are 0.43° for BaF_2_, 0.58° for CaF_2_, 0.44° for B-sil glass, and 0.35° for BK7 (Figure 2d). Although BK7 gives the narrowest resonance, its small ΔθSPR limits its usefulness for the present sensing design. CaF_2_ gives the largest angular response, but its resonance is broader than those obtained with BaF_2_ and B-sil glass. B-sil glass provides a balanced baseline response, combining a moderate angular shift of 5.77°, sensitivity enhancement of 8.97%, attenuation of 4.91%, and FWHM of 0.44°.

Based on these results, B-sil glass was selected as the prism material for the subsequent optimization steps. This selection does not imply that B-sil is universally superior to the other prisms. Rather, it provides a suitable compromise between angular response, resonance definition, and compatibility with the proposed multilayer architecture. The B-sil-supported Al configuration was therefore retained as the baseline structure for the subsequent Al, Al_2_O_3_, and WS_2_ optimization stages.

### 3.2. Effect of Aluminum Thickness on the SPR Response of the B-Sil/Al Platform

After selecting B-sil glass as the prism material, the thickness of the Al plasmonic layer was varied to define the baseline metal film for the SPR platform. The refractive-index values reported in Table 2 were used for the B-sil prism, water reference medium, and sensing-medium baseline. For the metallic layer, the complex refractive index of Al at λ = 633 nm was taken as 0.0778 + 5.8535i [54]. The tested structures are described in Table S2, while the corresponding optical response metrics are summarized in Table 4. The Al thickness was evaluated at 45, 50, 60, and 70 nm under the same angular interrogation conditions used in the prism-selection step. The sensing medium was kept at the baseline milk refractive index to isolate the effect of the metal-layer thickness on the resonance profile.

**Figure 3:**
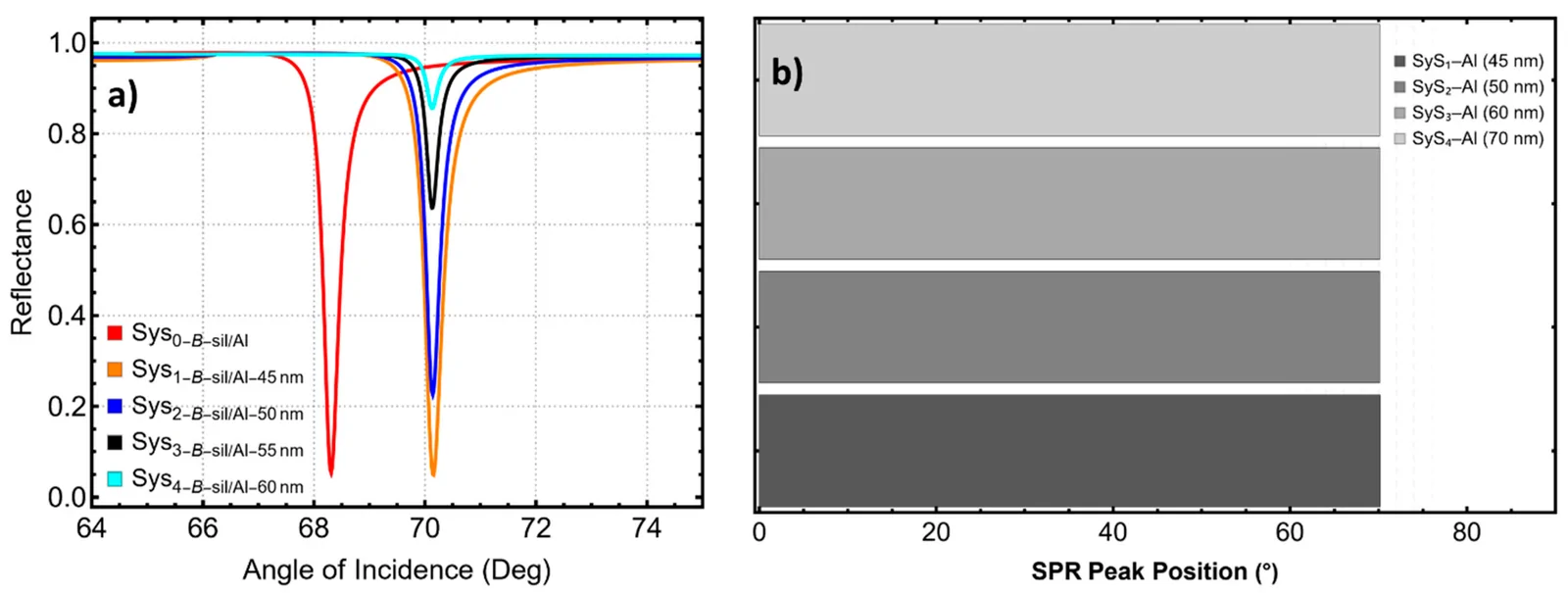
Aluminum-thickness-dependent SPR response of the B-sil-supported Al platform: (**a**) angular reflectance profiles for 45, 50, 60, and 70 nm Al layers and (**b**) corresponding SPR peak positions.

Figure 3a shows that changing the Al thickness modifies both the depth and width of the SPR dip. At 45 nm, the reflectance minimum is well defined and occurs near 70.15°. Increasing the thickness to 50 nm preserves a similar resonance position, with the SPR peak located at 70.14°. For 60 and 70 nm, the resonance position remains close to 70.12°, indicating that the angular location of the SPR minimum is only weakly affected once the Al film exceeds 50 nm. This limited angular displacement is also observed in Figure 3b and Table 4, where Δθ decreases slightly from 1.84° at 45 nm to 1.81° at 60–70 nm.

**Figure 4:**
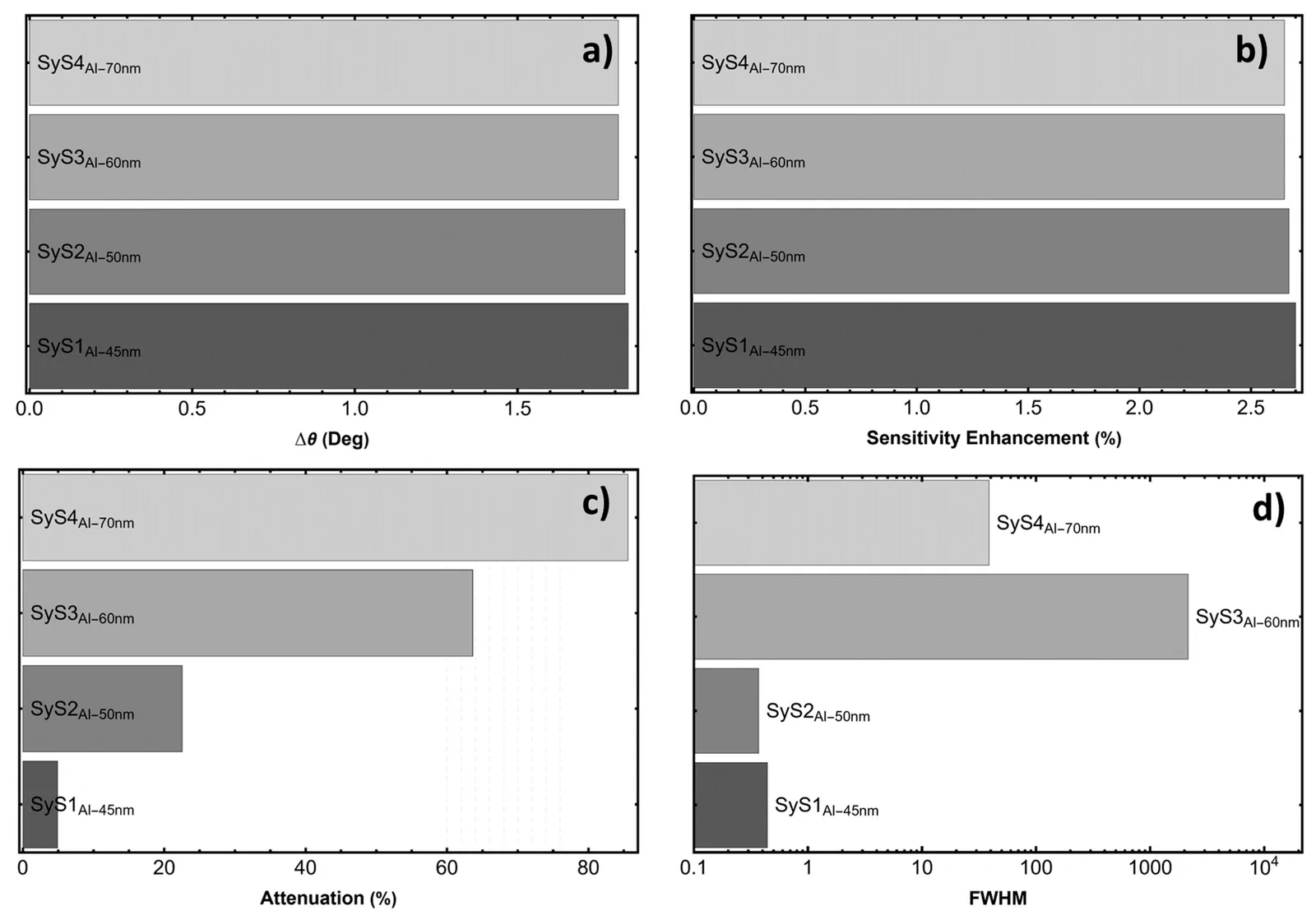
Aluminum-thickness-dependent sensing metrics of the B-sil-supported Al platform: (**a**) resonance-angle shift, Δθ_SPR_, (**b**) sensitivity enhancement, (**c**) attenuation, and (**d**) FWHM.

The sensitivity enhancement follows the same modest trend. As shown in Figure 4b, the enhancement decreases from 2.70% for the 45 nm Al layer to 2.65% for the 60 and 70 nm cases. This small variation indicates that the angular response alone is not sufficient to select the Al thickness. The attenuation and FWHM must also be considered because they describe the strength and definition of the resonance dip. Table 4 shows that attenuation increases from 4.91% at 45 nm to 22.55% at 50 nm, 63.63% at 60 nm, and 85.60% at 70 nm. This trend is also shown in Figure 4c and reflects stronger damping of the reflected signal as the Al layer becomes thicker.

The FWHM response is less uniform. The 45 and 50 nm films give narrow resonance profiles, with FWHM values of 0.44° and 0.37°, respectively. At 60 nm, the FWHM increases to 2153.04°, which indicates a poorly defined resonance profile under the numerical conditions used. The 70 nm film gives a lower FWHM than the 60 nm case but remains broader than the 45 and 50 nm films, with a value of 38.60°. This broadening suggests that thicker Al films reduce the sharpness of the angular resonance, even though they increase attenuation.

The 70 nm Al layer was retained for the next stage of the multilayer design. This choice was not defined by a single performance parameter. The 45 and 50 nm films produced sharper resonance dips, whereas the 70 nm film provided the strongest attenuation and maintained a stable SPR peak position near 70.12°. The next design step introduces an Al_2_O_3_ dielectric coating and a WS_2_ interfacial layer; for that reason, the 70 nm Al film was used as the baseline metal layer to evaluate how the added layers reshape the resonance profile and modify the sensor response. This choice should be interpreted as a design condition for the proposed multilayer sequence rather than as a general optimum for all Al-based SPR sensors.

### 3.3. Al_2_O_3_ Thickness Selection in the B-Sil/Al SPR Platform

The Al_2_O_3_ coating was introduced after fixing the B-sil/Al platform to examine how a dielectric overlayer modifies the plasmonic response. The structures used in this stage are defined in Table S3. The optical parameters are given in Table S4; the B-sil, water, and sensing-medium refractive indices follow the values reported in Table 2, the Al layer was fixed at 70 nm using a complex refractive index of 0.0778 + 5.8535i [54], and Al_2_O_3_ was modeled with a refractive index of 1.77 [57]. The coating thickness was varied from 2 to 30 nm while keeping the sensing medium at its baseline refractive index.

**Figure 5:**
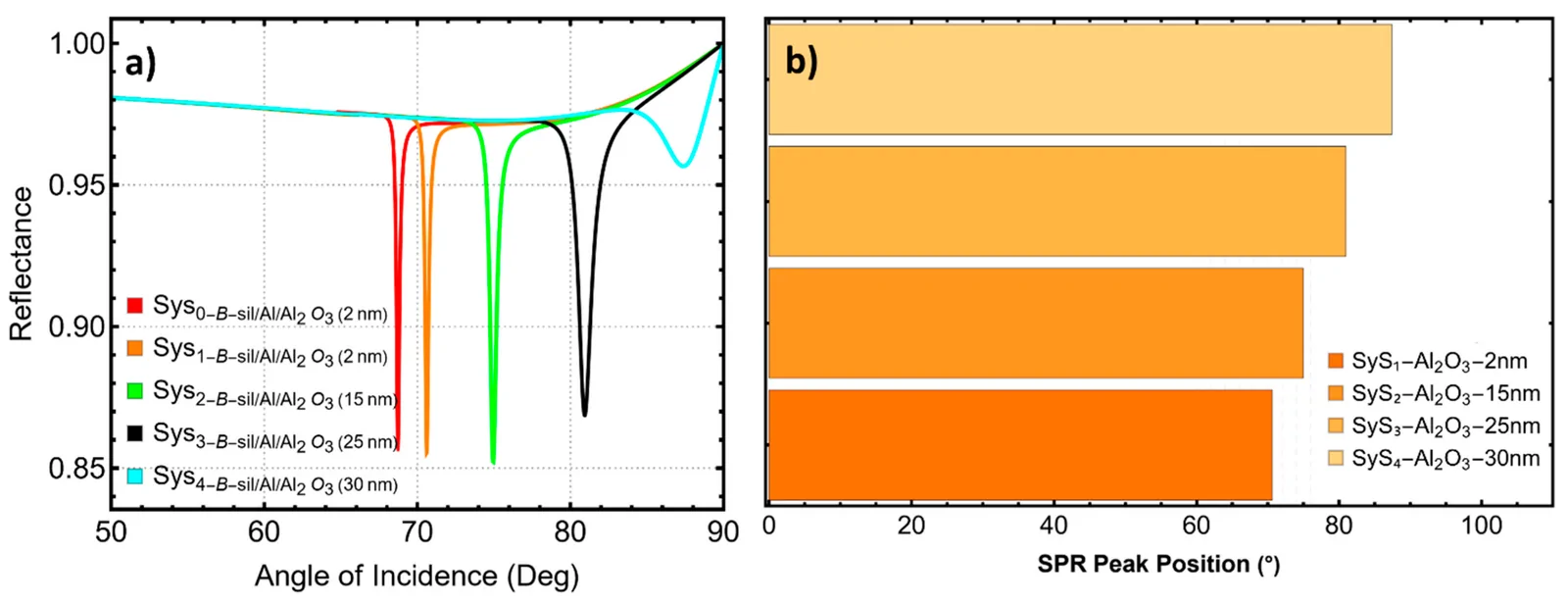
Al_2_O_3_-thickness-dependent SPR response of the B-sil/Al/Al_2_O_3_ platform: (**a**) angular reflectance profiles for 2, 15, 25, and 30 nm Al_2_O_3_ layers and (**b**) corresponding SPR peak positions.

The reflectance curves in Figure 5a show a progressive displacement of the resonance dip as the Al_2_O_3_ layer becomes thicker. With a 2 nm coating, the resonance appears at 70.60°, which remains close to the response of the uncoated Al platform. At 15 nm, the dip shifts to 74.95°. A further increase to 25 nm moves the resonance to 80.94°, while the 30 nm coating places the resonance at 87.38° (Figure 5b and Table 5). This trend indicates that the Al_2_O_3_ layer is not acting only as a passive protective film; it changes the optical phase condition of the multilayer and moves the SPR mode toward higher incidence angles.

**Figure 6:**
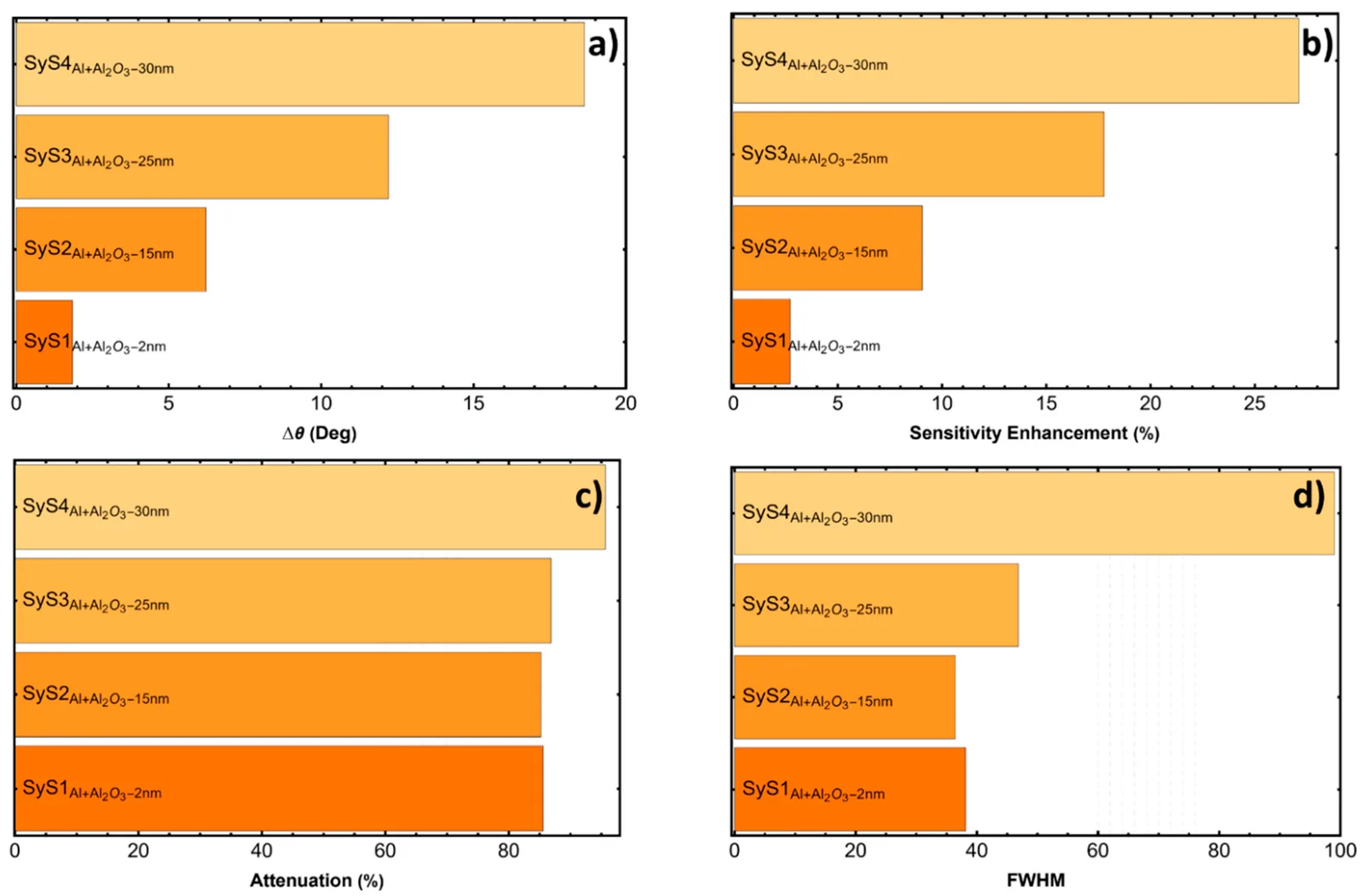
Al_2_O_3_-thickness-dependent sensing metrics of the B-sil/Al/Al_2_O_3_ platform: (**a**) resonance-angle shift, Δθ_SPR_, (**b**) sensitivity enhancement, (**c**) attenuation, and (**d**) FWHM.

The increase in Δθ_SPR_ follows this angular displacement. Table 5 reports Δθ_SPR_ values of 1.87°, 6.22°, 12.21°, and 18.64° for Al_2_O_3_ thicknesses of 2, 15, 25, and 30 nm, respectively. The sensitivity enhancement also increases across the same thickness range, from 2.72% to 27.12%, as shown in Figure 6a,b. This behavior is consistent with a stronger contribution of the dielectric layer to the evanescent-field distribution near the sensing interface. In practical terms, the thicker coatings give a larger angular displacement, but this gain must be interpreted together with the resonance profile.

The attenuation values remain within a relatively high range for the four Al_2_O_3_-coated structures. The 2, 15, and 25 nm layers give attenuation values of 85.54%, 85.22%, and 86.86%, respectively, while the 30 nm layer increases attenuation to 95.66% (Figure 6c and Table 5). The FWHM response shows the trade-off more clearly. The 15 nm coating gives the lowest FWHM among the tested Al_2_O_3_ cases, with 36.44°, followed by 2 nm with 38.11° and 25 nm with 46.84°. At 30 nm, the FWHM increases to 99.01° (Figure 6d), indicating that the resonance becomes much broader when the dielectric layer is too thick for the selected angular range.

The 25 nm Al_2_O_3_ layer was selected for the next stage because it gives a workable compromise between angular displacement and resonance definition. Compared with 2 and 15 nm, the 25 nm coating provides a larger Δ*θ_SPR_* and sensitivity enhancement. Compared with 30 nm, it avoids shifting the resonance too close to the high-angle edge of the scan and keeps the FWHM markedly lower. This condition gave Δ*θ_SPR_* = 12.21°, sensitivity enhancement = 17.76%, attenuation = 86.86%, and FWHM = 46.84° (Table 5). The B-sil/Al/Al_2_O_3_ platform with 70 nm Al and 25 nm Al_2_O_3_ was therefore used for the subsequent evaluation of the WS_2_ interfacial layer.

### 3.4. Selection of the WS_2_ Interfacial Layer in the B-Sil/Al/Al_2_O_3_ SPR Platform

The final material-selection step examined the effect of adding a 2D interfacial layer to the B-sil/Al/Al_2_O_3_ platform. The evaluated structures are defined in Table S5, and the optical constants and layer thicknesses used in the simulation are reported in Table S6. The platform retained the previously selected B-sil prism, 70 nm Al layer, and 25 nm Al_2_O_3_ coating. The tested 2D materials were black phosphorus (BP), molybdenum disulfide (MoS_2_), molybdenum diselenide (MoSe_2_), and tungsten disulfide (WS_2_), using refractive indices of 3.50 + 0.01i, 5.0805 + 1.1723i, 4.62 + 1.0063i, and 4.90 + 0.3124i, respectively, at λ = 633 nm (Table S6) [54]. Water and the sensing-medium baseline were kept at the refractive-index values used in the previous stages.

**Figure 7:**
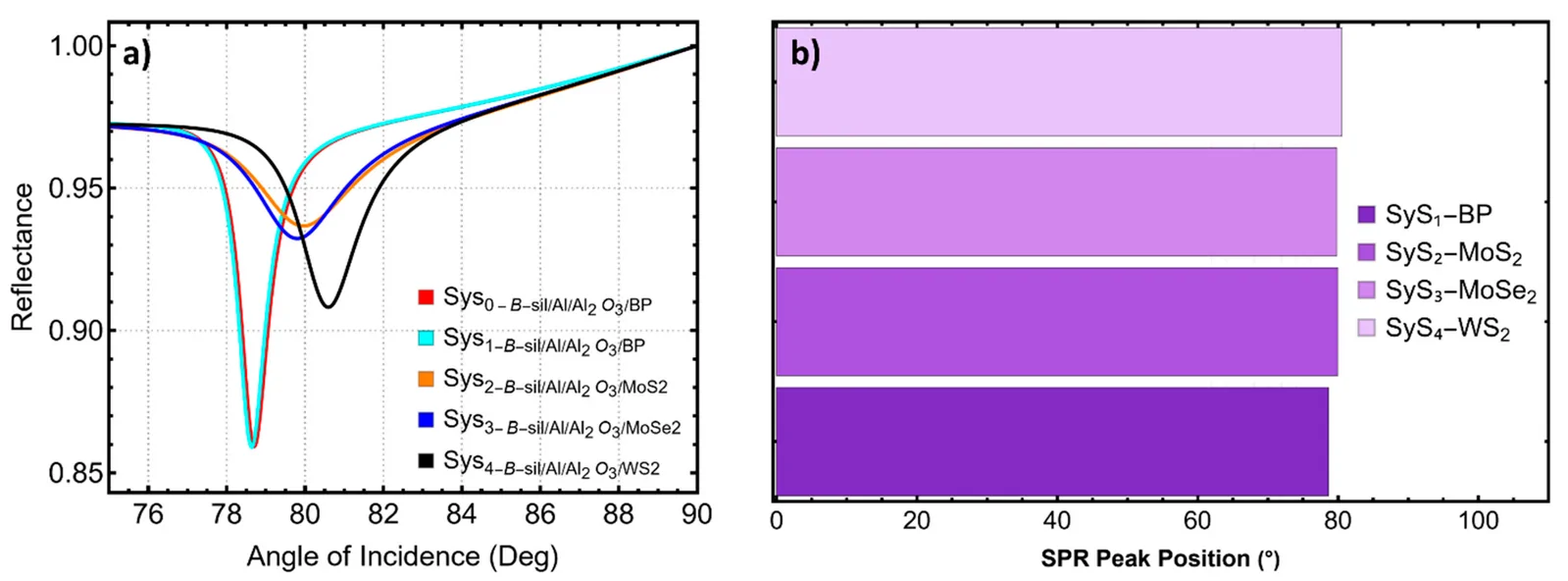
Effect of the 2D interfacial material on the angular SPR response of the B-sil/Al/Al_2_O_3_ platform: (**a**) reflectance curves for BP, MoS_2_, MoSe_2_, and WS_2_ interfacial layers and (**b**) corresponding SPR peak positions.

Figure 7a shows that the 2D layer changes the depth and angular position of the resonance dip. The BP-coated system gives the resonance at 78.64°, close to the reference condition of the coated platform. The TMDC-containing structures shift the resonance toward higher angles, with peak positions of 79.95° for MoS_2_, 79.80° for MoSe_2_, and 80.59° for WS_2_ (Figure 7b and Table 6). This displacement indicates that the interfacial material contributes to the phase condition of the multilayer and alters the optical interaction at the sensing boundary.

**Figure 8:**
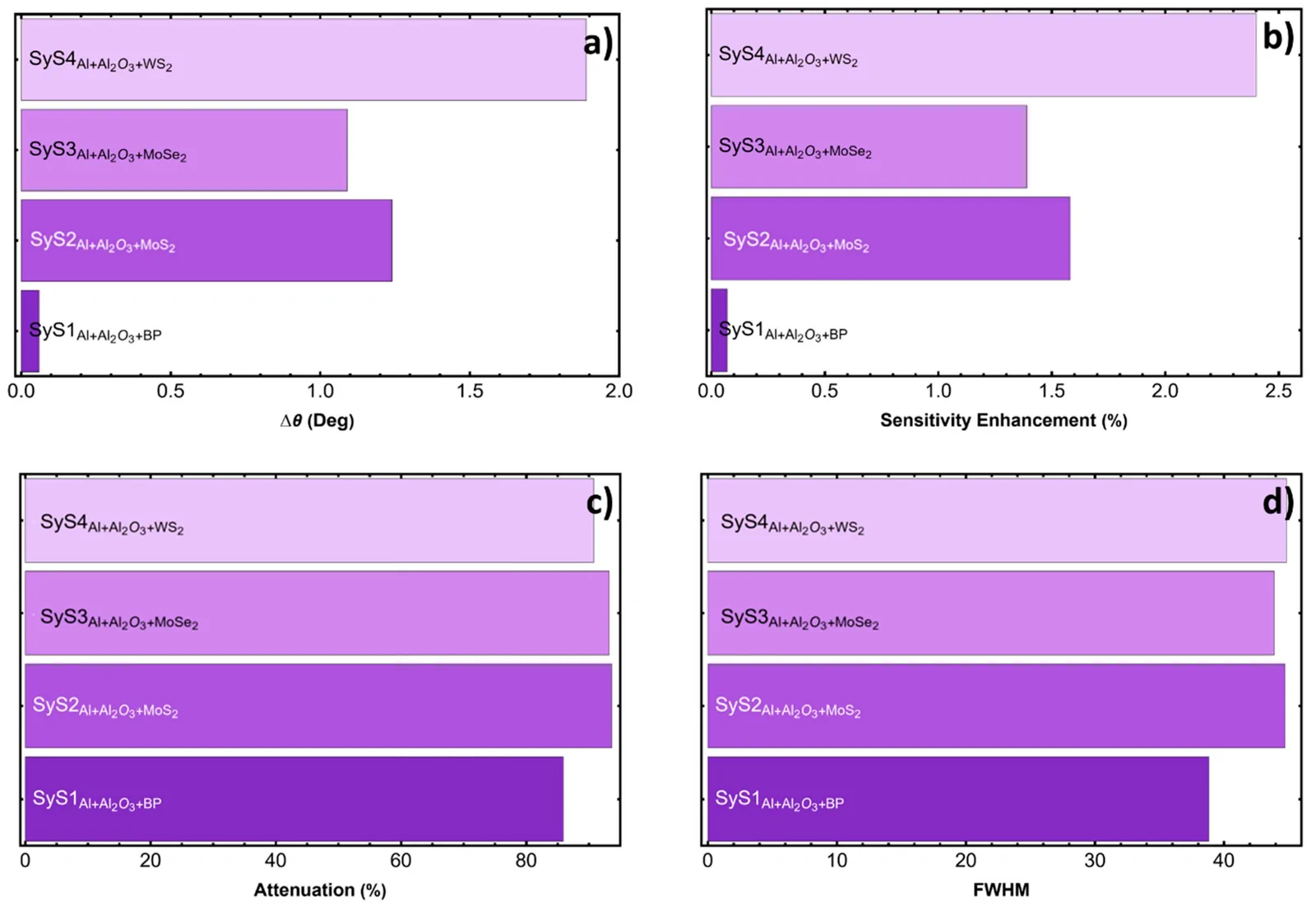
Effect of the 2D interfacial material on the sensing metrics of the B-sil/Al/Al_2_O_3_ platform: (**a**) resonance-angle shift, Δθ_SPR_, (**b**) sensitivity enhancement, (**c**) attenuation, and (**d**) FWHM.

The angular-shift data in Table 6 show that BP produces a small Δθ_SPR_ of 0.06°, whereas MoS_2_ and MoSe_2_ produce larger shifts of 1.24° and 1.09°, respectively. WS_2_ gives the highest value among the tested 2D layers, with Δθ_SPR_ = 1.89°. The same tendency is observed for sensitivity enhancement in Figure 8b, where WS_2_ reaches 2.40%, followed by MoS_2_ at 1.58%, MoSe_2_ at 1.39%, and BP at 0.07%. These differences are consistent with the distinct optical constants of the 2D layers, which modify the evanescent-field interaction near the analyte interface.

Attenuation remains high for the four interfacial materials, as shown in Figure 8c. Table 6 reports attenuation values of 85.91% for BP, 93.66% for MoS_2_, 93.22% for MoSe_2_, and 90.81% for WS_2_. MoS_2_ and MoSe_2_ give slightly higher attenuation than WS_2_, but their angular shifts and sensitivity enhancements are lower. The FWHM values also remain within a comparable range for the TMDC-based structures. BP gives the lowest FWHM, 38.82°, while MoS_2_, MoSe_2_, and WS_2_ give 44.72°, 43.89°, and 44.84°, respectively (Figure 8d and Table 6). The WS_2_ case therefore introduces only a modest broadening relative to the other TMDCs while giving the largest angular response.

WS_2_ was selected as the interfacial layer for the final sensing configuration. This selection reflects the balance between angular displacement, sensitivity enhancement, attenuation, and resonance width rather than a single isolated metric. At the selected condition, the B-sil/Al/Al_2_O_3_/WS_2_/sensing-medium structure produced an SPR peak at 80.59°, Δθ_SPR_ = 1.89°, sensitivity enhancement = 2.40%, attenuation = 90.81%, and FWHM = 44.84° (Table 6). These values indicate that WS_2_ provides a suitable interfacial condition for the following H_2_O_2_-dependent refractive-index analysis while preserving a measurable resonance profile within the simulated angular range.

### 3.5. B-Sil/Al/Al_2_O_3_/WS_2_ Sensor Under Different H_2_O_2_ Concentration Levels in Milk

The response of the selected B-sil/Al/Al_2_O_3_/WS_2_ structure was examined using milk media containing increasing H_2_O_2_ concentrations. The simulated conditions comprised the baseline sensing medium, denoted SM, and six H_2_O_2_-containing samples, denoted C1–C6. The concentration and refractive-index values assigned to each condition are reported in Table 7. H_2_O_2_ concentration increased from 0.00% for SM to 14.29% for C6, while the refractive index increased from 1.34550 to 1.34686. The multilayer configuration consisted of a B-sil prism, a 70 nm Al layer, a 25 nm Al_2_O_3_ coating, and a 0.80 nm WS_2_ interfacial layer. Angular interrogation was performed at λ = 633 nm.

**Figure 9:**
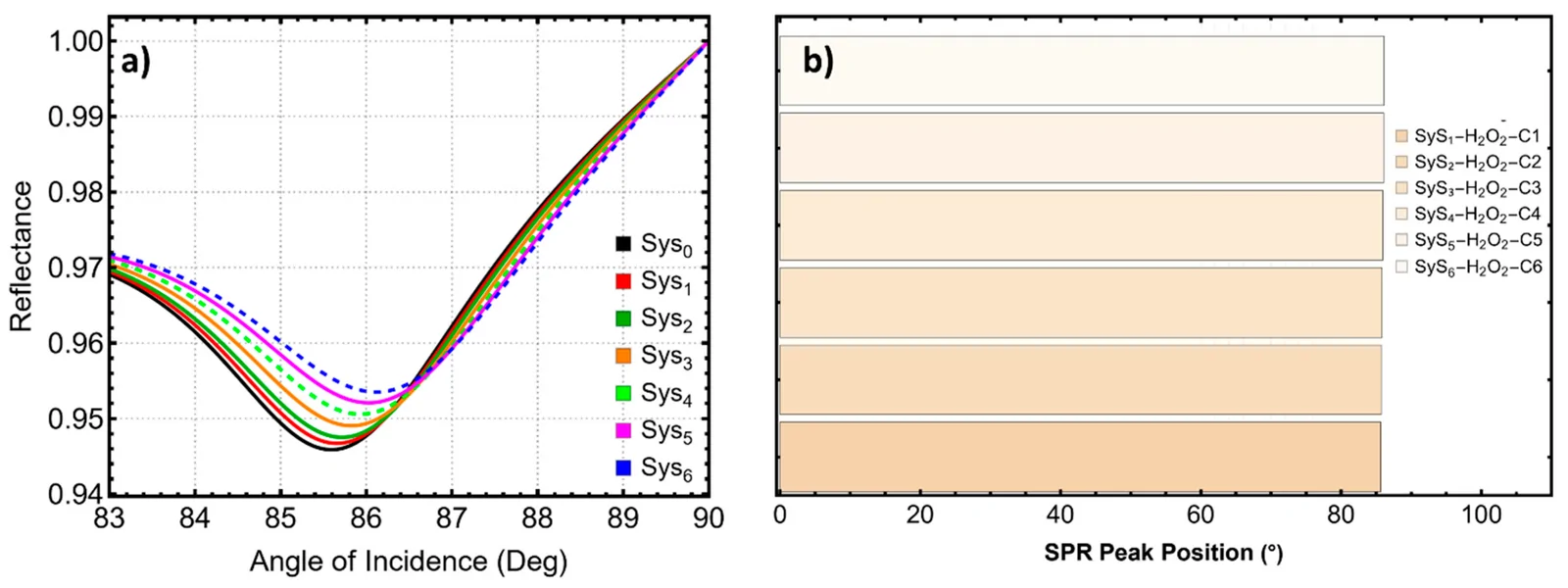
H_2_O_2_-dependent angular SPR response of the optimized B-sil/Al/Al_2_O_3_/WS_2_ sensor: (**a**) reflectance profiles for the baseline and H_2_O_2_-containing milk media and (**b**) SPR peak positions.

Figure 9a shows that the reflectance profiles retain a similar shape across the simulated concentration range, while the resonance minimum shifts toward higher incidence angles as the H_2_O_2_ concentration and refractive index increase. The corresponding peak positions are shown in Figure 9b and summarized in Table 8. The resonance angle changes from 85.65° for C1 to 86.11° for C6, giving a total displacement of 0.46° between the lowest and highest simulated H_2_O_2_ conditions.

**Figure 10:**
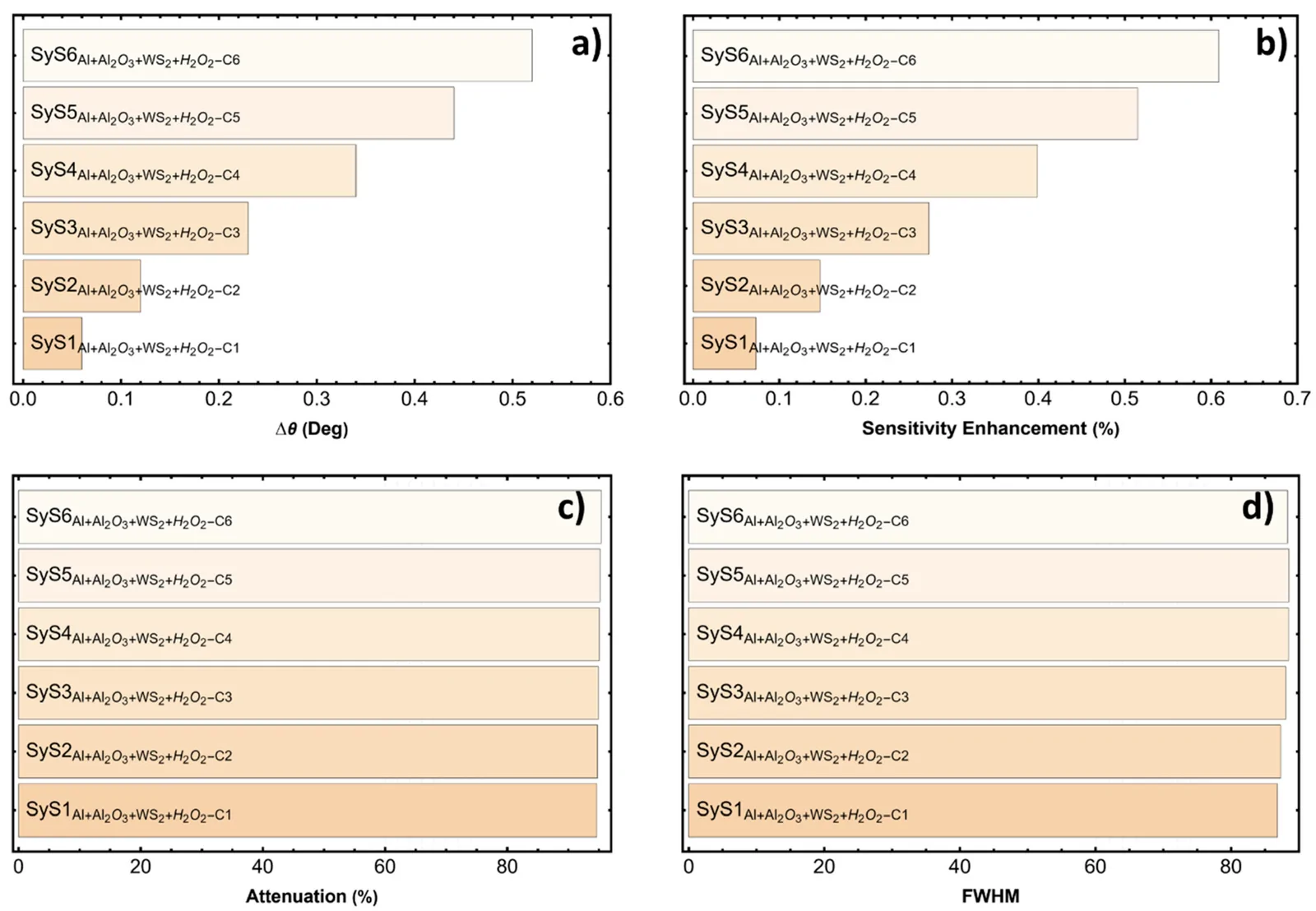
H_2_O_2_-dependent response metrics of the optimized B-sil/Al/Al_2_O_3_/WS_2_ sensor: (**a**) resonance-angle shift, Δθ_SPR_, (**b**) sensitivity enhancement, (**c**) attenuation, and (**d**) FWHM.

The resonance-angle shift was calculated relative to the baseline sensing medium. As reported in Table 8 and shown in Figure 10a, Δθ_SPR_ increases from 0.06° for C1 to 0.52° for C6. This monotonic response follows the refractive-index increase listed in Table 7, from 1.34566 for C1 to 1.34686 for C6. The sensitivity enhancement follows the same trend, increasing from 0.073% to 0.609%, as shown in Figure 10b and Table 8.

The calculated response is governed by bulk refractive-index variation rather than selective chemical recognition of H_2_O_2_. The model therefore represents an optical screening approach in which changes in milk composition modify the phase-matching condition of the SPR mode. Experimental measurements would be required to determine whether the angular shift can be distinguished from refractive-index changes caused by other milk constituents or adulterants.

The attenuation changes only slightly across the concentration series, ranging from 94.67% to 95.35%, as reported in Table 8 and shown in Figure 10c. This limited variation indicates that increasing H_2_O_2_ concentration affects the angular position of the resonance more strongly than its minimum reflectance. The FWHM values range from 86.80° to 88.53° (Table 8 and Figure 10d), indicating a broad resonance profile under the present numerical conditions. These width values should be verified from the two half-depth crossing angles before FWHM-dependent performance metrics are interpreted.

The selected B-sil/Al/Al_2_O_3_/WS_2_ structure therefore produces a monotonic angular response to the simulated H_2_O_2_-induced refractive-index variation. Across C1–C6, the resonance angle and Δθ_SPR_ increase with H_2_O_2_ concentration, while attenuation remains within a narrow interval. These angular shifts provide the basis for calculating refractive-index sensitivity after the FWHM extraction and the associated width-dependent metrics have been verified.

### 3.6. H_2_O_2_ Concentration-Dependent Sensing Performance of the Optimized B-Sil/Al/Al_2_O_3_/WS_2_ Sensor

**Figure 11:**
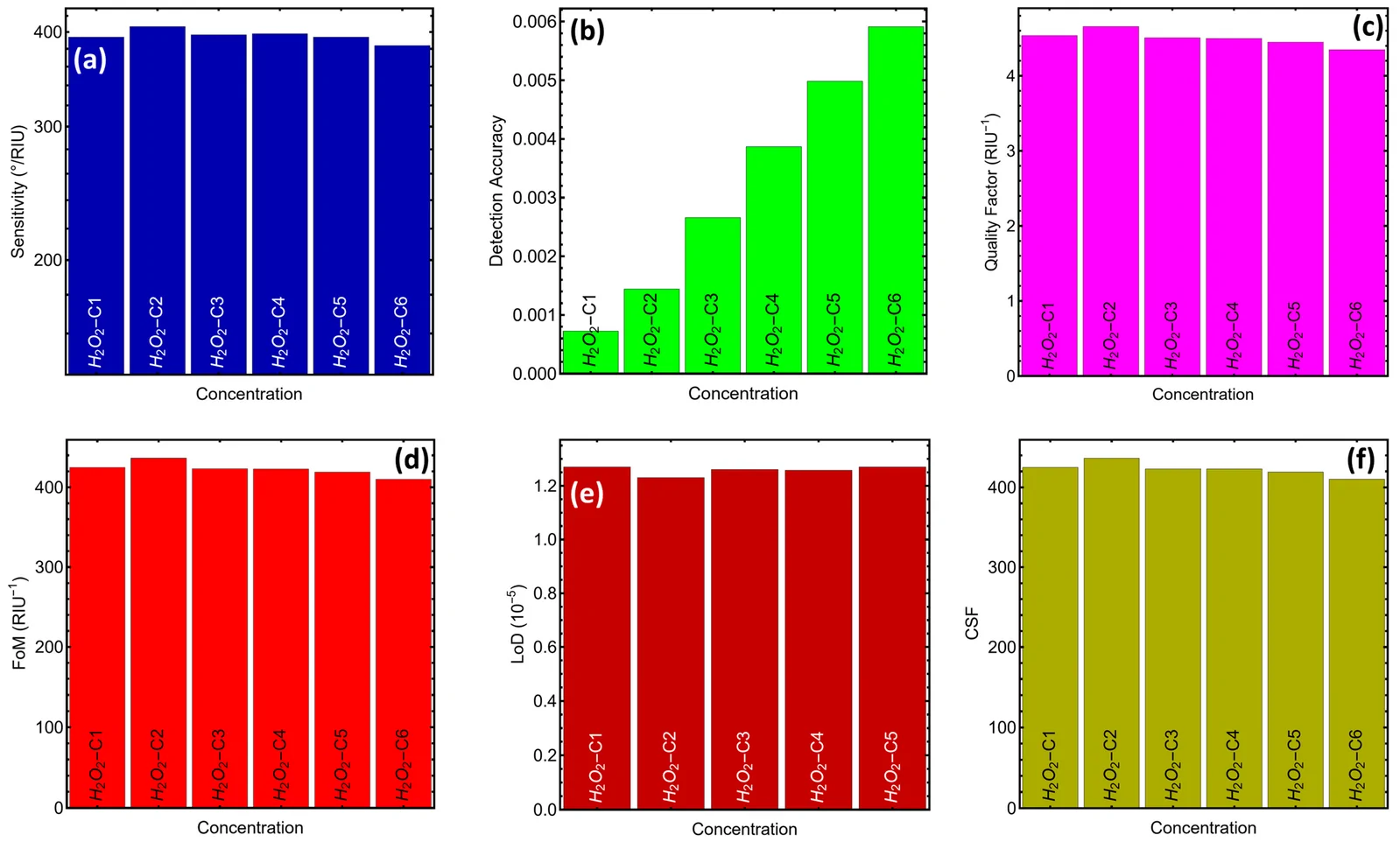
Concentration-dependent performance of the selected B-sil/Al/Al_2_O_3_/WS_2_ SPR configuration under H_2_O_2_-associated refractive-index variations in milk: (**a**) angular sensitivity, (**b**) detection accuracy, (**c**) quality factor, (**d**) figure of merit, (**e**) estimated limit of detection, and (**f**) combined sensitivity factor.

The concentration-dependent performance of the selected B-sil/Al/Al_2_O_3_/WS_2_ configuration was evaluated from the angular shifts calculated for six modeled H_2_O_2_ cases in milk. The corresponding metrics are reported in Table 9 and Figure 11. This analysis extends the angular response discussed in Section 3.5 by expressing the resonance shifts in terms of angular sensitivity, detection accuracy, quality factor, figure of merit, estimated limit of detection, and combined sensitivity factor.

The angular sensitivity remains within a narrow range, from 383.82 to 406.45°/RIU (Table 9). Figure 11a shows that H_2_O_2_-C2 gives the highest sensitivity, while lower values are obtained for the remaining cases. This variation indicates that the calculated angular response is not strictly linear over the full simulated refractive-index range. The lower sensitivity values at higher H_2_O_2_ levels can be associated with the smaller incremental resonance displacement relative to the refractive-index change used in the sensitivity calculation. Despite this variation, all six cases remain within the same order of magnitude, indicating a comparable angular response over the evaluated refractive-index range.

Detection accuracy shows a different pattern. As shown in Figure 11b, DA increases from 0.00072 for H_2_O_2_-C1 to 0.00591 for H_2_O_2_-C6. This behavior is linked to the FWHM-dependent definition of DA used in the calculations. The higher DA values obtained for the later H_2_O_2_ cases reflect the corresponding resonance widths. These values must therefore be interpreted together with the FWHM results reported in the previous section, since DA is derived directly from the calculated resonance width rather than from an independently measured experimental resolution.

The quality factor and figure of merit show a similar distribution. The QF reaches its highest value at H_2_O_2_-C2, with 4.65 RIU^−1^, and subsequently decreases to 4.34 RIU^−1^ at H_2_O_2_-C6 (Figure 11c and Table 9). The FoM also reaches its maximum at H_2_O_2_-C2, with 436.48 RIU^−1^, and decreases to 410.10 RIU^−1^ at H_2_O_2_-C6 (Figure 11d). The combined sensitivity factor follows the same pattern, with a maximum of 436.56 at H_2_O_2_-C2 and a minimum of 410.18 at H_2_O_2_-C6 (Figure 11f). Within the present numerical dataset, H_2_O_2_-C2 gives the largest calculated values of sensitivity, QF, FoM, and CSF.

The estimated limit of detection varies only slightly, ranging from 1.230 × 10^−5^ to 1.302 × 10^−5^ (Table 9). Figure 11e shows the lowest estimated LoD at H_2_O_2_-C2, with higher values for the other evaluated cases. This limited variation reflects the relatively narrow range of calculated angular sensitivity. The minimum estimated LoD therefore occurs at the same H_2_O_2_ case that gives the largest sensitivity, QF, FoM, and CSF.

Taken together, Table 9 and Figure 11 characterize the optical response of the selected B-sil/Al/Al_2_O_3_/WS_2_ configuration for the six H_2_O_2_-associated refractive-index cases evaluated in milk. H_2_O_2_-C2 gives the largest calculated sensitivity, QF, FoM, and CSF and the lowest estimated LoD within the modeled dataset. At higher H_2_O_2_ levels, DA is larger while the sensitivity-related metrics are lower. These results describe the numerical response of the multilayer structure at the evaluated points and should not be interpreted as evidence of chemical selectivity toward H_2_O_2_. Further numerical refinement should focus on the resonance profile and its influence on FWHM-dependent metrics before experimental implementation is considered.

### 3.7. Electric Field Distribution Analysis

**Figure 12:**
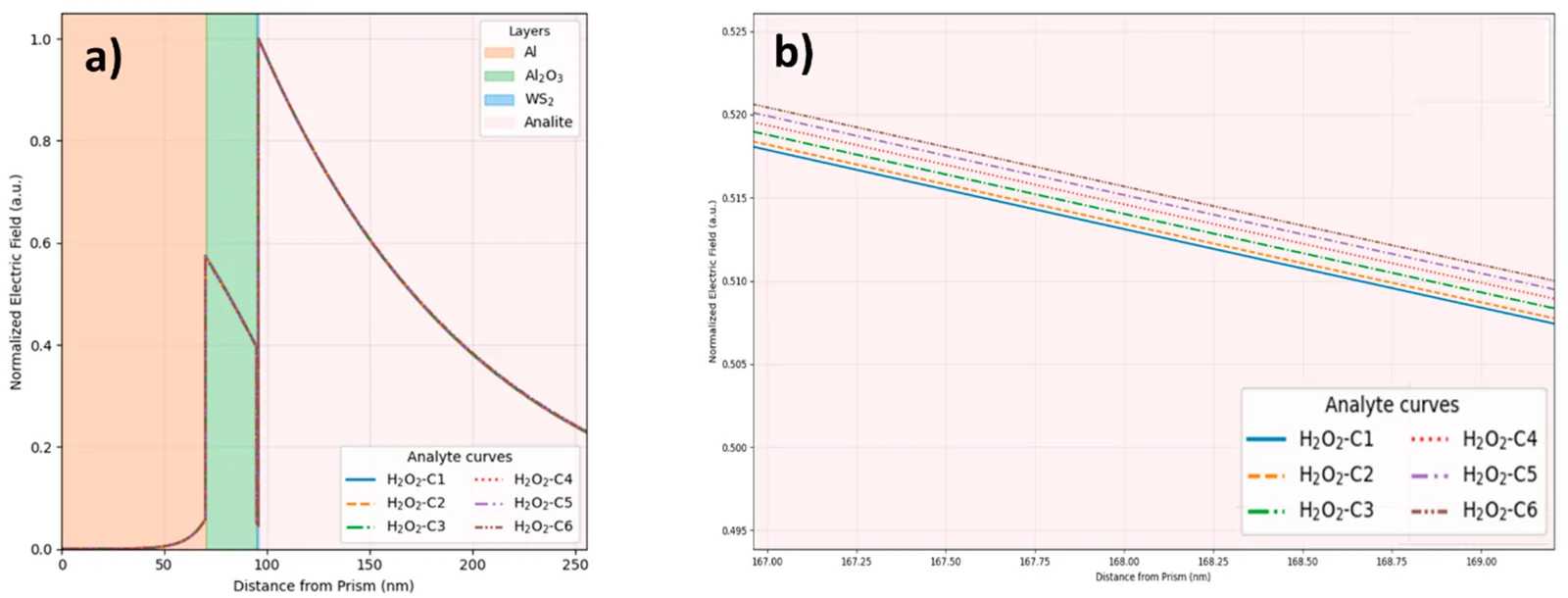
Normalized electric-field distribution of the optimized B-sil/Al/Al_2_O_3_/WS_2_ SPR sensor for H_2_O_2_ detection in milk: (**a**) field profile across the multilayer stack and analyte region and (**b**) magnified field decay within the sensing medium for the modeled H_2_O_2_ concentration levels.

The electric-field profile was examined to identify how the optical field is distributed across the optimized multilayer structure and how it extends into the H_2_O_2_-containing sensing medium. Figure 12a shows the normalized electric-field distribution from the Al layer to the analyte region. The field remains low inside the Al film, increases near the Al/Al_2_O_3_ region, and reaches its maximum close to the WS_2_/analyte interface. This localization is consistent with SPR excitation, where the evanescent field is concentrated near the metal–dielectric boundary and decays into the external medium.

The selected structure contains three functional regions before the analyte: Al as the plasmonic layer, Al_2_O_3_ as the dielectric coating, and WS_2_ as the interfacial layer. In Figure 12a, the field increases through the dielectric region and changes sharply near the WS_2_/analyte boundary. This behavior indicates that the sensing response is governed by the optical field present at the outer interface, where the milk/H_2_O_2_ medium is located. Because the evanescent field penetrates into the analyte region, changes in the refractive index of the milk medium can modify the resonance condition and produce the angular shifts discussed in Section 3.5 and Section 3.6.

Figure 12b presents a magnified view of the field decay inside the analyte region. The curves associated with H_2_O_2_-C1 to H_2_O_2_-C6 show similar decay profiles, with small separation among concentration levels. This result is expected because the modeled H_2_O_2_ concentrations introduce relatively small refractive-index changes in the sensing medium. The field amplitude decreases with distance from the WS_2_/analyte interface, confirming that the strongest optical interaction occurs near the sensor surface. This near-interface sensitivity supports the use of WS_2_ as the terminal layer in the proposed configuration.

The field distribution also helps explain the moderate concentration-dependent changes reported in Table 7 and Table 8. The resonance angle shifts systematically with H_2_O_2_ concentration, but the field profiles remain close to each other because the refractive-index contrast between adjacent concentration levels is small. The sensor therefore responds mainly through angular displacement rather than through a large change in field amplitude. This behavior is appropriate for a theoretical refractive-index SPR sensor and indicates that future experimental work should control the analyte–surface contact region carefully, since the sensing signal is concentrated near the WS_2_/milk interface.

### 3.8. Comparison with Reported SPR Studies

The performance of the B-sil/Al/Al_2_O_3_/WS_2_ configuration was compared with reported SPR systems applied to milk adulteration, preservative detection, or milk-composition analysis, as summarized in Table 10. The comparison includes CaF_2_/Cu/BaTiO_3_/graphene for preservative detection in milk [31], ZnSe/Au/graphene/BlueP-MoS_2_ for milk adulteration analysis [58], CaF_2_/Ag/BP for milk adulteration detection [59], and Ag/barium gallium sulfide/cadmium gallium sulfide/BlueP-MoS_2_ for fat-concentration analysis in milk [60]. These studies provide an application-relevant benchmark because they use SPR transduction in milk-related sensing and report at least one of the performance metrics considered in the present work.

The calculated angular sensitivity of the present configuration ranges from 383.82 to 406.45°/RIU across the six simulated H_2_O_2_ conditions. This range exceeds the 219°/RIU reported for the CaF_2_/Cu/BaTiO_3_/graphene structure [31] and the 51.38°/RIU reported for the ZnSe/Au/graphene/BlueP-MoS_2_ structure [58]. It is also close to the 433.02°/RIU reported for the Ag/barium gallium sulfide/cadmium gallium sulfide/BlueP-MoS_2_ platform [60]. The CaF_2_/Ag/BP structure shows the highest sensitivity in this comparison, reaching 574°/RIU [59]. The present configuration therefore does not maximize angular sensitivity among the reported systems, but it maintains values above 380°/RIU using an Al-based plasmonic layer combined with Al_2_O_3_ and WS_2_.

The quality factor of the present configuration ranges from 4.34 to 4.65 RIU^−1^. These values are lower than those reported for CaF_2_/Cu/BaTiO_3_/graphene, CaF_2_/Ag/BP, and Ag/barium gallium sulfide/cadmium gallium sulfide/BlueP-MoS_2_, which show QF values of 102.34, 200, and 310.12 RIU^−1^, respectively [31,59,60]. This difference is associated with the broad resonance profiles obtained for the Al/Al_2_O_3_/WS_2_ multilayer. The QF comparison therefore indicates that resonance-width reduction remains an important aspect for further refinement of the configuration.

Detection accuracy increases from 0.00072 to 0.00591 across the simulated H_2_O_2_ conditions. These values are lower than the DA values of 0.47 and 0.223 reported for the CaF_2_/Cu/BaTiO_3_/graphene and CaF_2_/Ag/BP structures, respectively [31,59]. The ZnSe/Au/graphene/BlueP-MoS_2_ and Ag/barium gallium sulfide/cadmium gallium sulfide/BlueP-MoS_2_ studies did not report DA values [58,60]. This behavior is consistent with the FWHM dependence discussed in Section 3.6 and indicates that the present configuration is more favorable in angular sensitivity than in resonance sharpness.

Among the six simulated conditions, H_2_O_2_-C2 gives the largest calculated sensitivity and QF, with values of 406.45°/RIU and 4.65 RIU^−1^, respectively, while its DA is 0.00144. H_2_O_2_-C6 gives the highest DA but the lowest sensitivity and QF within the modeled series. Within this numerical comparison, the B-sil/Al/Al_2_O_3_/WS_2_ configuration can be interpreted as a sensitivity-oriented theoretical SPR architecture for evaluating H_2_O_2_-associated refractive-index variations in milk. Further work should focus on narrowing the resonance profile, experimentally validating the H_2_O_2_-associated refractive-index response in real milk samples, and incorporating an appropriate selective recognition or filtering strategy for practical implementation.

### 3.9. Proposed Fabrication Pathway for the SPR Sensor

**Figure 13:**
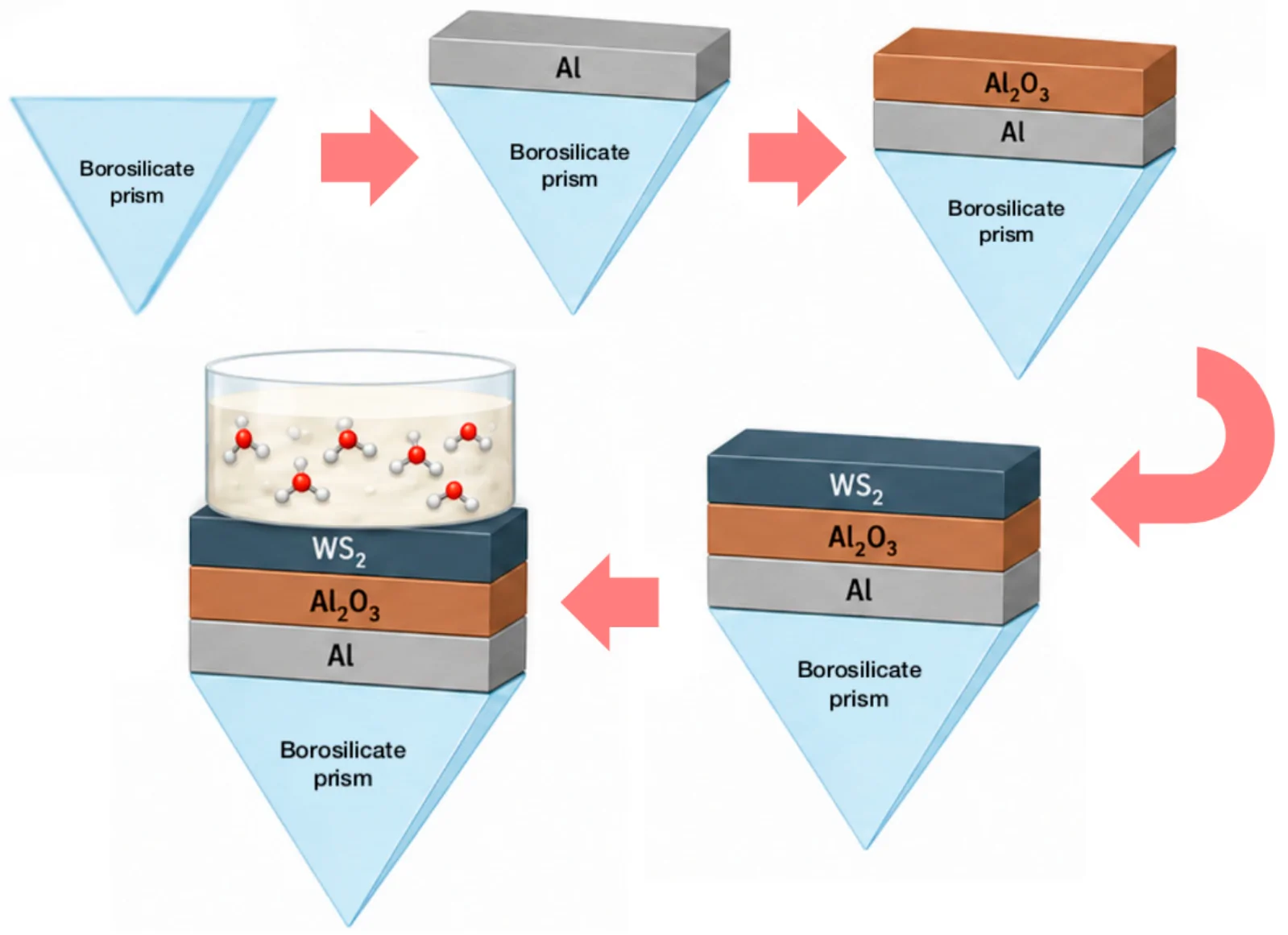
Conceptual fabrication pathway proposed for future development of the SPR sensor, showing sequential assembly of the prism substrate, Al layer, Al_2_O_3_ coating, WS_2_ layer, and milk/H_2_O_2_ sensing medium.

Figure 13 presents a conceptual fabrication pathway for the proposed SPR sensor. This scheme is intended to illustrate the planned assembly sequence for future experimental development and should not be interpreted as evidence of device fabrication or validation in the present study. The proposed route begins with the optical prism substrate, followed by deposition of the Al plasmonic layer. An Al_2_O_3_ coating is then introduced over the Al film to act as a dielectric and protective layer. The WS_2_ layer is subsequently placed at the sensing interface to modify the evanescent field and support stronger interaction with the analyte-containing medium. In the final configuration, the milk sample containing H_2_O_2_ is brought into contact with the WS_2_ surface, where concentration-dependent refractive-index changes are expected to induce shifts in the SPR angle.

This assembly route provides a practical basis for translating the numerical design into an experimental platform. Future validation should include controlled thin-film deposition, thickness and roughness characterization, optical alignment under angular interrogation, and testing with milk samples containing known H_2_O_2_ concentrations. Additional experiments will be required to evaluate repeatability, stability of the Al/Al_2_O_3_ interface, WS_2_ layer uniformity, matrix effects from real milk samples, and selectivity against other possible adulterants or interfering compounds.

### 3.10. Limitations and Scalability of the Proposed Sensor

The present study is theoretical, so the results should be interpreted within the assumptions of the transfer matrix model. The simulations considered flat, continuous, and optically uniform layers with fixed refractive indices and thicknesses. In a fabricated sensor, film roughness, thickness deviations, interface quality, Al oxidation, and WS_2_ coverage may change the resonance angle, attenuation, and FWHM. These nonidealities may produce deviations between the calculated SPR response and the response of a fabricated multilayer, particularly in resonance position, minimum reflectance, and resonance linewidth.

The sensing response was modeled through refractive-index changes associated with H_2_O_2_ in milk. This approach characterizes the theoretical optical response of the B-sil/Al/Al_2_O_3_/WS_2_ configuration, but it does not include a selective chemical-recognition layer. Real milk contains proteins, lipids, lactose, salts, and other possible adulterants that may also modify the refractive index. Experimental validation is therefore required to confirm selectivity toward H_2_O_2_.

A second limitation is the broad resonance profile of the selected configuration. Although the configuration shows calculated sensitivity values between 383.82 and 406.45°/RIU, the QF and DA remain lower than some reported SPR platforms. Future optimization should focus on reducing the FWHM while preserving the H_2_O_2_-dependent angular shift.

From a scalability perspective, the multilayer architecture is compatible, in principle, with planar thin-film fabrication because it uses an Al plasmonic layer, an Al_2_O_3_ protective coating, and a WS_2_ interfacial layer. Future work should fabricate the multilayer stack, verify thickness and surface uniformity, calibrate the sensor with reference liquids, and test real milk samples containing known H_2_O_2_ concentrations. Selectivity studies against other adulterants will also be needed before practical application.

## 4. Conclusions

This theoretical study evaluated the angular SPR response of a B-sil/Al/Al_2_O_3_/WS_2_ multilayer configuration to refractive-index variations associated with H_2_O_2_ in milk under TM-polarized illumination at λ = 633 nm. The multilayer architecture was established through sequential evaluation of prism material, Al thickness, Al_2_O_3_ thickness, and 2D interfacial material. This procedure led to the selection of B-sil glass, 70 nm Al, 25 nm Al_2_O_3_, and 0.80 nm WS_2_ as the working configuration for analysis of the H_2_O_2_-associated refractive-index range.

Across the simulated H_2_O_2_ cases, the resonance angle increased from 85.65° to 86.11° as the refractive index of the sensing medium changed. The calculated angular sensitivity ranged from 383.82 to 406.45°/RIU. The best balance among the evaluated metrics was obtained for H_2_O_2_-C2, with S = 406.45°/RIU, QF = 4.65 RIU^−1^, FoM = 436.48 RIU^−1^, LoD = 1.23 × 10^−5^, and CSF = 436.56. The electric-field analysis also showed field localization near the WS_2_/sensing-medium interface, consistent with the angular response obtained from the multilayer model.

The systematic assessment of the B-sil/Al/Al_2_O_3_/WS_2_ architecture provides a design-oriented evaluation of this material combination for angular SPR monitoring of H_2_O_2_-associated refractive-index changes in milk. Comparison with reported SPR systems for milk-related sensing showed a comparable angular sensitivity range, while the broad resonance profile limited QF and detection accuracy. These results describe the theoretical optical behavior of the proposed structure and do not demonstrate chemical selectivity toward H_2_O_2_. Experimental implementation would require fabrication of the multilayer stack, calibration with reference liquids and real milk samples, and incorporation of an appropriate selective filtering or recognition strategy to distinguish H_2_O_2_ from other constituents or adulterants that may produce similar refractive-index variations.

## Figures and Tables

**Table 1:** Optical and geometrical parameters of the final B-sil/Al/Al_2_O_3_/WS_2_ SPR configuration at λ = 633 nm.

Layer/Medium	Refractive Index	Thickness (nm)	Ref.
B-sil prism	1.4701	-	[42]
Al	0.0778 + 5.8535i	70	[50]
Al_2_O_3_	1.7700	25	[53]
WS_2_	4.9000 + 0.3124i	0.80	[50]
Water medium (H_2_O)	1.3300	-	[51]
Sensing medium (SM) (baseline)	1.3455	-	[52]

**Table 2:** Refractive indices of prism materials and reference media used for the SPR configuration at λ = 633 nm.

Material	Refractive Index	Ref.
BaF_2_	1.4733	[54]
CaF_2_	1.4329	[54]
B-sil	1.4701	[46]
BK7	1.5151	[54]
Water medium (H_2_O)	1.3300	[55]
Sensing medium (SM) (baseline)	1.3455	[56]

**Table 3:** SPR response metrics obtained for the Al-based sensor using different prism materials and the sensing-medium baseline.

Code	SPR Peak Position	∆θ (Deg)	Sensitivity Enhancement (%)	Attenuation (%)	FWHM
Sys_1-BaF2_	69.81	5.43	8.43	4.93	0.43
Sys_2-CaF2_	74.77	10.39	16.14	3.90	0.58
Sys_3-B-sil_	70.15	5.77	8.97	4.91	0.44
Sys_4-BK7_	65.88	1.15	2.32	6.14	0.35

**Table 4:** SPR response metrics obtained for the B-sil/Al/sensing-medium platform at different Al thicknesses.

Code	Thickness (nm)	SPR Peak Position	∆θ (Deg)	Sensitivity Enhancement (%)	Attenuation (%)	FWHM
Sys_1-Al-45nm_	45	70.15	1.84	2.70	4.91	0.44
Sys_2-Al-50nm_	50	70.14	1.83	2.67	22.55	0.37
Sys_3-Al-60nm_	60	70.12	1.81	2.65	63.63	2153.04
Sys_4-Al-70nm_	70	70.12	1.81	2.65	85.60	38.60

**Table 5:** SPR response metrics obtained for the B-sil/Al/Al_2_O_3_/sensing-medium platform at different Al_2_O_3_ thicknesses.

Code	Thickness (nm)	SPR Peak Position	∆θ (Deg)	Sensitivity Enhancement (%)	Attenuation (%)	FWHM
Sys_1-Al+Al2O3-2nm_	2	70.60	1.87	2.72	85.54	38.11
Sys_2-Al+Al2O3-15nm_	15	74.95	6.22	9.05	85.22	36.44
Sys_3-Al+Al2O3-25nm_	25	80.94	12.21	17.76	86.86	46.84
Sys_4-Al+Al2O3-30nm_	30	87.38	18.64	27.12	95.66	99.01

**Table 6:** SPR response metrics of the B-sil/Al/Al_2_O_3_/sensing-medium platform after incorporating different 2D interfacial materials.

Code	SPR Peak Position	∆θ (Deg)	Sensitivity Enhancement (%)	Attenuation (%)	FWHM
Sys_1-B-sil/Al/Al2O3/BP/SM_	78.64	0.06	0.07	85.91	38.82
Sys_2-B-sil/Al/Al2O3/MoS2/SM_	79.95	1.24	1.58	93.66	44.72
Sys_3-B-sil/Al/Al2O3/MoSe2/SM_	79.80	1.09	1.39	93.22	43.89
Sys_4-B-sil/Al/Al2O3/WS2/SM_	80.59	1.89	2.40	90.81	44.84

**Table 7:** Concentration vs Refractive index.

Code	Concentration (%)	RI
SM	0.0000	1.34550
C1	1.63934	1.34566
C2	3.22581	1.34581
C3	6.25000	1.34609
C4	9.09091	1.34636
C5	11.76471	1.34662
C6	14.28571	1.34686

**Table 8:** SPR response metrics of the optimized B-sil/Al/Al_2_O_3_/WS_2_ sensor for the simulated H_2_O_2_ concentration levels in milk.

Code	SPR Peak Position	∆θ (Deg)	Sensitivity Enhancement (%)	Attenuation (%)	FWHM
Sys_1-B-sil/Al/Al2O3/WS2/H2O2-C1_	85.65	0.06	0.073	94.67	86.80
Sys_2-B-sil/Al/Al2O3/WS2/H2O2-C2_	85.71	0.12	0.147	94.75	87.30
Sys_3-B-sil/Al/Al2O3/WS2/H2O2-C3_	85.82	0.23	0.273	94.90	88.03
Sys_4-B-sil/Al/Al2O3/WS2/H2O2-C4_	85.93	0.34	0.399	95.05	88.45
Sys_5-B-sil/Al/Al2O3/WS2/H2O2-C5_	86.03	0.44	0.515	95.20	88.53
Sys_6-B-sil/Al/Al2O3/WS2/H2O2-C6_	86.11	0.52	0.609	95.35	88.30

**Table 9:** Concentration-dependent performance metrics of the selected B-sil/Al/Al_2_O_3_/WS_2_ SPR configuration under H_2_O_2_-associated refractive-index variations in milk.

Concentration	*S* (°/RIU)	DA	QF (*RIU*^−1^)	FoM (*RIU*^−1^)	LoD (10^−5^)	CSF
H_2_O_2_-C1	393.75	0.00072	4.53	424.88	1.269	424.96
H_2_O_2_-C2	406.45	0.00144	4.65	436.48	1.230	436.56
H_2_O_2_-C3	396.61	0.00265	4.50	423.07	1.260	423.15
H_2_O_2_-C4	397.67	0.00386	4.49	422.88	1.257	422.96
H_2_O_2_-C5	393.75	0.00498	4.44	418.97	1.269	419.05
H_2_O_2_-C6	383.82	0.00591	4.34	410.10	1.302	410.18

**Table 10:** Comparison of reported SPR-based sensors for milk adulteration detection and the proposed B-sil/Al/Al_2_O_3_/WS_2_ sensor under the simulated H_2_O_2_ concentration conditions.

Ref.	SPR Structure	Sensitivity (°/RIU)	QF (RIU^−1^)	DA
[31]	CaF_2_/Cu/BaTiO_3_/graphene/SM	219	102.34	0.47
[58]	ZnSe/Au/graphene/BlueP-MoS_2_/SM	51.38	No reported	No reported
[59]	CaF_2_/Ag/BP/SM	574	200	0.223
[60]	Ag/Barium gallium sulfide/Cadmium gallium sulfide/BlueP-MoS_2_/SM	433.02	310.12	No reported
This study	B-sil/Al/Al_2_O_3_/WS_2_/H_2_O_2_-C1	393.75	4.53	0.00072
This study	B-sil/Al/Al_2_O_3_/WS_2_/H_2_O_2_-C2	406.45	4.65	0.00144
This study	B-sil/Al/Al_2_O_3_/WS_2_/H_2_O_2_-C3	396.61	4.50	0.00265
This study	B-sil/Al/Al_2_O_3_/WS_2_/H_2_O_2_-C4	397.67	4.49	0.00386
This study	B-sil/Al/Al_2_O_3_/WS_2_/H_2_O_2_-C5	393.75	4.44	0.00498
This study	B-sil/Al/Al_2_O_3_/WS_2_/H_2_O_2_-C6	383.82	4.34	0.00591

## Data Availability

Data presented in this study are available upon reasonable request from the corresponding author.

## Supplementary Materials

The following supporting information can be downloaded at: https://www.mdpi.com/article/10.3390/bios16090494/s1, Figure S1: Validation of a Numerical Modeling Approach Using Experimental Results from Ref. [38]; Table S1: Code definitions and layer descriptions for the prism-selection study; Table S2: Code definitions and layer descriptions for the aluminum-thickness optimization study; Table S3: Code definitions and layer descriptions for the Al_2_O_3_-thickness optimization study; Table S4: Optical parameters used for the borosilicate/Al/Al_2_O_3_/sensing-medium structure at λ = 633 nm; Table S5: Code definitions and layer descriptions for the 2D interfacial-material study; Table S6: Optical parameters used for the borosilicate/Al/Al_2_O_3_/2D-material/sensing-medium structures at λ = 633 nm; Table S7: Code definitions and layer descriptions for the H_2_O_2_ concentration study; Table S8: Optical parameters used for the optimized borosilicate/Al/Al_2_O_3_/WS_2_/sensing medium structure at λ = 633 nm.
