# Supplementary material for: Design and Performance Analysis of an SPR Sensor for Milk Adulteration Monitoring
Source: Biosensors (Basel). 2026 Sep 4;16(9):494. doi: 10.3390/bios16090494 (PMC13604231; doi:10.3390/bios16090494)
Supplement: Supplementary file 1 [file biosensors-16-00494-s001.zip › biosensors-4495650-supplementary.pdf]

SUPPLEMENTARY INFORMATION: Design and Performance Analysis of an SPR Sensor for Milk Adulteration Monitoring

John Germán Vera Luzuriaga[1,*], Marco Guevara[2], Diana Coello-Fiallos[3], Cristian Vacacela Gomez[4,*]

[1] Departamento de Ciencias de la Energía y Mecánica, Universidad de las Fuerzas Armadas ESPE, Latacunga EC050150, Ecuador
[2] Facultad de Mecánica, Escuela Superior Politécnica de Chimborazo (ESPOCH), Riobamba 060155, Ecuador
[3] Carrera de Física, Grupo GIMA, Facultad de Ciencias, Escuela Superior Politécnica de Chimborazo (ESPOCH), Riobamba 060155, Ecuador
[4] Universidad Ecotec, Km. 13.5 Samborondón, Samborondón, EC092302, Ecuador

* Correspondence: jgvera4@espe.edu.ec (J.G.V.L.) & cristianisaac.vacacelagomez@fis.unical.it (C.V.G.)

S1. Additional performance metrics

The quality factor was calculated using Eq. (S1):

$$QF = \frac{S_{RI}}{FWHM}\ (RIU^{-1}) \tag{S1}$$

Because Eq. (S1) is equivalent to the figure of merit reported in Eq. (10) of the main manuscript, QF was retained only as a supplementary metric to avoid redundancy in the main text.

The combined sensitivity factor was calculated using Eq. (S2):

$$CSF = \frac{S_{RI} \times (R_{max} - R_{min})}{FWHM}\ (RIU^{-1}) \tag{S2}$$

where R_{max} is the maximum reflectance before resonance and R_{min} is the minimum reflectance at resonance.

S2. Numerical settings

All calculations were performed under TM-polarized illumination at λ=633 nm. The angular reflectance curves were generated using 5×10^4 sampling points. The resonance angle was identified from the minimum of the reflectance curve, and the FWHM was extracted from the angular width of the resonance dip at half depth. The same numerical settings were used for all H_2O_2 concentrations to allow direct comparison among sensing conditions.

Supplementary Figure

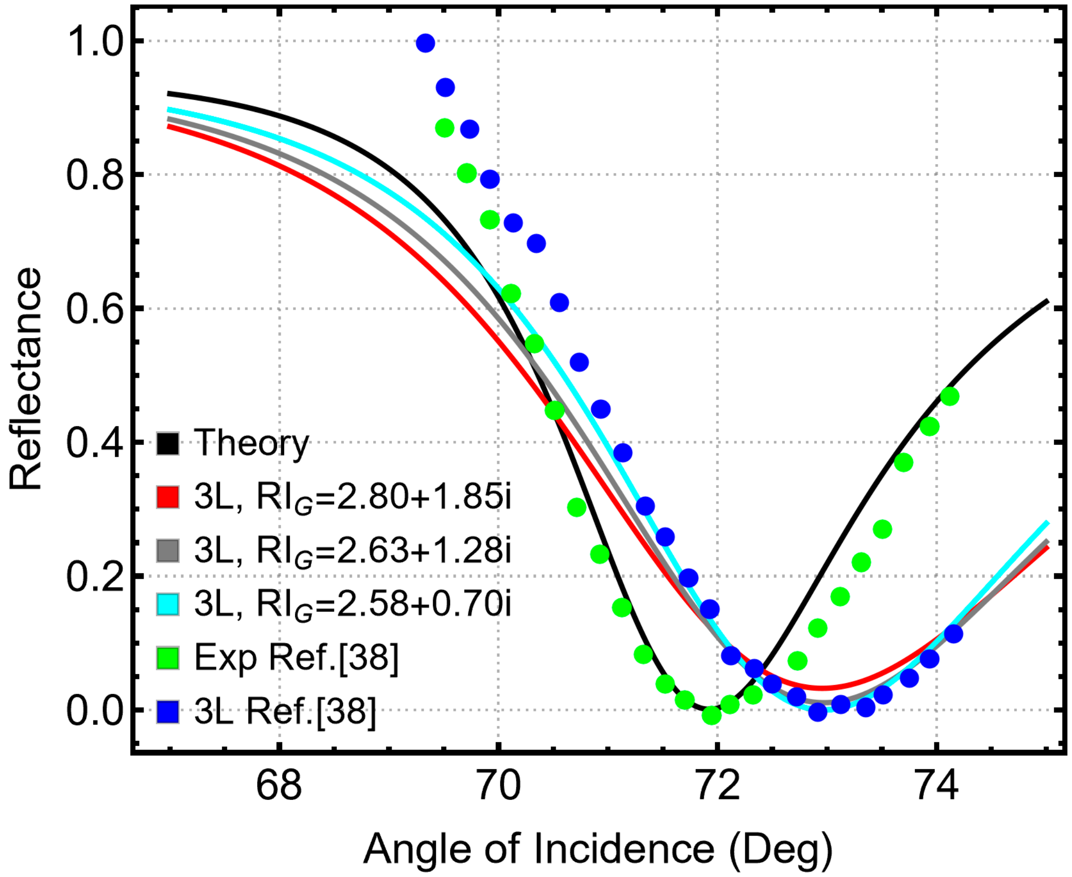

Figure S1. Validation of a Numerical Modeling Approach Using Experimental Results from Ref. [38]

Supplementary Tables

Table S1. Code definitions and layer descriptions for the prism-selection study.

Code	Full Name	Short Name
$Sys_{0\text{-}Bk7}$	BK7/Aluminium/Water	BK7/Al/H_2O
$Sys_{1\text{-}BaF2}$	Barium Fluoride/Aluminium/Sensing Medium	BaF_2/Al/SM
$Sys_{2\text{-}CaF2}$	Calcium Fluoride /Aluminium/Sensing Medium	CaF_2/Al/SM
$Sys_{3\text{-}B\text{-}sil}$	Borosilicate /Aluminium/Sensing Medium	B-sil/Al/SM
$Sys_{4\text{-}BK7}$	BK7/Aluminium/Sensing Medium	BK7/Al/SM

Table S2. Code definitions and layer descriptions for the aluminum-thickness optimization study.

Code	Full Name	Short Name
$Sys_{0\text{-}Al\text{-}45nm}$	B-sil/Aluminum-45 nm/Water	B-sil/Al-$_{45nm}$/H_2O
$Sys_{1\text{-}Al\text{-}45nm}$	B-sil/Aluminum-45 nm/Sensing Medium	B-sil/Al-$_{45nm}$/SM
$Sys_{2\text{-}Al\text{-}50nm}$	B-sil/Aluminum-50 nm/Sensing Medium	B-sil/Al-$_{50nm}$/SM
$Sys_{3\text{-}Al\text{-}60nm}$	B-sil/Aluminum-60 nm/Sensing Medium	B-sil/Al-$_{60nm}$/SM

$Sys_{4\text{-}Al\text{-}70nm}$	B-sil/Aluminum-70 nm/Sensing Medium	B-sil/Al-$_{70nm}$/SM

Table S3. Code definitions and layer descriptions for the Al_2O_3-thickness optimization study.

Code	Full Name	Short Name
$Sys_{0\text{-}Al+Al_2O_3\text{-}2nm}$	B-sil/Aluminum/Aluminum Oxide-2 nm/Water	B-sil/Al/Al_2O_3 - $_{2\ nm}$/H_2O
$Sys_{1\text{-}Al+Al_2O_3\text{-}2nm}$	B-sil/Aluminum/Aluminum Oxide-2 nm/Sensing Medium	B-sil/Al/Al_2O_3 - $_{2\ nm}$/SM
$Sys_{2\text{-}Al+Al_2O_3\text{-}15nm}$	B-sil/Aluminum/Aluminum Oxide-15 nm/Sensing Medium	B-sil/Al/Al_2O_3 - $_{15\ nm}$/SM
$Sys_{3\text{-}Al+Al_2O_3\text{-}25nm}$	B-sil/Aluminum/Aluminum Oxide-25 nm/Sensing Medium	B-sil/Al/Al_2O_3 - $_{25\ nm}$/SM
$Sys_{4\text{-}Al+Al_2O_3\text{-}30nm}$	B-sil/Aluminum/Aluminum Oxide-30 nm/Sensing Medium	B-sil/Al/Al_2O_3 - $_{30\ nm}$/SM

Table S4. Optical parameters used for the borosilicate/Al/Al_2O_3/sensing-medium structure at λ = 633 nm.

Material	Refractive Index	Thickness (nm)	Ref.
B-sil	1.4701	--	[46]
Aluminum	0.0778 + 5.8535i	70	[54]
Aluminum Oxide (Al_2O_3)	1.77	2.00	[55]
Water medium (H_2O)	1.330	--	[56]
Sensing medium (SM) (baseline)	1.3455	--	[57]

Table S5. Code definitions and layer descriptions for the 2D interfacial-material study.

Code	Full Name	Short Name
$Sys_{0\text{-}B\text{-}sil/Al/Al_2O_3/BP/H_2O}$	B-sil/Aluminum/Aluminum Oxide/Black Phosphorus/Water	B-sil/Al/Al_2O_3/BP/H_2O
$Sys_{1\text{-}B\text{-}sil/Al/Al_2O_3/BP/SM}$	B-sil/Aluminum/Aluminum Oxide/Black Phosphorus/Sensing Medium	B-sil/Al/Al_2O_3/BP/SM
$Sys_{2\text{-}B\text{-}sil/Al/Al_2O_3/MoS_2/SM}$	B-sil/Aluminum/Aluminum Oxide/Molybdenum Disulfide/Sensing Medium	B-sil/Al/Al_2O_3/MoS_2/SM
$Sys_{3\text{-}B\text{-}sil/Al/Al_2O_3/MoSe_2/SM}$	B-sil/Aluminum/Aluminum Oxide/Molybdenum Diselenide/Sensing Medium	B-sil/Al/Al_2O_3/$MoSe_2$/SM
$Sys_{4\text{-}B\text{-}sil/Al/Al_2O_3/WS_2/SM}$	B-sil/Aluminum/Aluminum Oxide/Tungsten Disulfide/Sensing Medium	B-sil/Al/Al_2O_3/WS_2/SM

Table S6. Optical parameters used for the borosilicate/Al/Al_2O_3/2D-material/sensing-medium structures at λ = 633 nm.

Material	Refractive Index	Thickness (nm)	Ref.
B-sil	1.4701	--	[46]
Aluminum	0.0778 + 5.8535i	70.0	[54]
Aluminum Oxide (Al_2O_3)	1.77	25.0	[55]
Black Phosphorus (BP)	3.5 + 0.01i	0.53	[54]
Molybdenum Disulfide (MoS_2)	5.0805 + 1.1723i	0.65	[54]
Molybdenum Diselenide ($MoSe_2$)	4.62 + 1.0063i	0.70	[54]
Tungsten Disulfide (WS_2)	4.90 + 0.3124i	0.80	[54]

Water medium (H_2O)	1.330	--	[56]
Sensing medium (SM) (baseline)	1.3455	--	[57]

Table S7. Code definitions and layer descriptions for the H_2O_2 concentration study.

Code	Full Name	Short Name
$Sys_{0\text{-B-sil/Al/Al}_2\text{O}_3\text{/WS}_2\text{/SM}}$	B-sil/Aluminum/Aluminum Oxide/Tungsten Disulfide/Sensing Medium	B-sil/Al/Al_2O_3/WS_2/SM
$Sys_{1\text{-B-sil/Al/Al}_2\text{O}_3\text{/WS}_2\text{/H}_2\text{O}_2\text{-C1}}$	B-sil/Aluminum/Aluminum Oxide/Tungsten Disulfide/Hydrogen Peroxide – Concentration 1	B-sil/Al/Al_2O_3/WS_2/H_2O_2-C1
$Sys_{2\text{-B-sil/Al/Al}_2\text{O}_3\text{/WS}_2\text{/H}_2\text{O}_2\text{-C2}}$	B-sil/Aluminum/Aluminum Oxide/Tungsten Disulfide/Hydrogen Peroxide – Concentration 2	B-sil/Al/Al_2O_3/WS_2/H_2O_2-C2
$Sys_{3\text{-B-sil/Al/Al}_2\text{O}_3\text{/WS}_2\text{/H}_2\text{O}_2\text{-C3}}$	B-sil/Aluminum/Aluminum Oxide/Tungsten Disulfide/Hydrogen Peroxide – Concentration 3	B-sil/Al/Al_2O_3/WS_2/H_2O_2-C3
$Sys_{4\text{-B-sil/Al/Al}_2\text{O}_3\text{/WS}_2\text{/H}_2\text{O}_2\text{-C4}}$	B-sil/Aluminum/Aluminum Oxide/Tungsten Disulfide/Hydrogen Peroxide – Concentration 4	B-sil/Al/Al_2O_3/WS_2/H_2O_2-C4
$Sys_{5\text{-B-sil/Al/Al}_2\text{O}_3\text{/WS}_2\text{/H}_2\text{O}_2\text{-C5}}$	B-sil/Aluminum/Aluminum Oxide/Tungsten Disulfide/Hydrogen Peroxide – Concentration 5	B-sil/Al/Al_2O_3/WS_2/H_2O_2-C5
$Sys_{6\text{-B-sil/Al/Al}_2\text{O}_3\text{/WS}_2\text{/H}_2\text{O}_2\text{-C6}}$	B-sil/Aluminum/Aluminum Oxide/Tungsten Disulfide/Hydrogen Peroxide – Concentration 6	B-sil/Al/Al_2O_3/WS_2/H_2O_2-C6

Table S8. Optical parameters used for the optimized borosilicate/Al/Al_2O_3/WS_2/sensing-medium structure at $\lambda = 633$ nm.

Material	Refractive Index	Thickness (nm)	Ref.
B-sil	1.4701	--	[46]
Aluminum	0.0778 + 5.8535i	70.0	[54]
Aluminum Oxide (Al_2O_3)	1.77	25.0	[33]
Tungsten Disulfide (WS_2)	4.90 + 0.3124i	0.80	[54]
Water medium (H_2O)	1.330	--	[56]
Sensing medium (SM) (baseline)	1.3455	--	[57]

Prompt - Scheme 1

At the center, depict a transparent borosilicate glass prism (light blue, semi-transparent, triangular prism) with realistic glass reflections and refraction. A red laser beam (633 nm He–Ne laser) enters from the left side, refracts inside the prism, reflects at the prism–metal interface, and exits toward an optical detector positioned on the right. Show the incident angle using a curved black arc labeled θ, together with a dashed vertical reference line through the prism.

Above the prism, display a multilayer sensing structure composed of perfectly aligned rectangular layers:

- Borosilicate prism (transparent light blue)
- Aluminum (Al) layer (metallic silver)
- Aluminum Oxide (Al_2O_3) layer (bronze/light brown dielectric)
- Tungsten Disulfide (WS_2) layer (dark blue-gray nanosheet)

Below the WS_2 layer, illustrate the sensing medium, represented as a transparent chamber filled with milk. The liquid should appear creamy-white with subtle texture and contain numerous water molecules (red oxygen atoms with two white hydrogen atoms) distributed throughout the medium to indicate the analyte. The sensing medium should be labeled Milk with H_2O_2.

On the left side, draw a realistic blue cylindrical laser module labeled:

- He–Ne Laser Source (633 nm)

On the right side, draw a realistic blue optical detector connected by a black cable and label it:

- Optical Detector

Along the right margin, include a clean vertical legend with horizontal leader lines identifying each layer in order:

- Borosilicate Prism
- Aluminum (Al)
- Aluminum Oxide (Al_2O_3)
- Tungsten Disulfide (WS_2)
- Sensing Medium (Milk with H_2O_2)
- Style Requirements
- Scientific graphical abstract
- Realistic 3D isometric perspective
- Vector-quality rendering
- Photorealistic material textures
- Soft ambient lighting
- Metallic brushed texture for Al
- Matte dielectric finish for Al_2O_3
- Layered nanosheet appearance for WS_2
- Transparent optical-quality glass prism
- Bright red laser path with subtle glow
- Crisp black typography using a professional serif or sans-serif scientific font
- Symmetrical composition
- Clean white background
- Minimalistic academic design
- High contrast
- Ultra-high resolution (4K–8K)
- Suitable for publication in Nature, ACS Nano, Sensors, Biosensors, Plasmonics, or MDPI journals

Prompt - Figure 13

The figure should show the sequential fabrication of the SPR sensing platform connected by large red arrows:

Step 1: A transparent inverted triangular borosilicate glass prism, light blue with subtle transparency, labeled Borosilicate prism.

Step 2: The same prism coated with a thin metallic Aluminum (Al) layer on the top surface, silver-gray rectangular film labeled Al.

Step 3: Add a thin Aluminum Oxide (Al_2O_3) dielectric layer above the aluminum. The Al_2O_3 layer should be orange-brown and labeled Al_2O_3. The stack now consists of:

- Borosilicate prism
- Al
- Al_2O_3

Step 4: Add a dark blue-gray WS_2 (Tungsten Disulfide) nanosheet above the Al_2O_3 layer, labeled WS_2. The multilayer stack becomes:

- Borosilicate prism
- Al
- Al_2O_3
- WS_2

Final Step: Place a transparent cylindrical container filled with a slightly cloudy liquid representing a milk sample directly on top of the WS_2 layer. Inside the liquid, show numerous small water molecules (red oxygen atoms with two white hydrogen atoms) and suspended particles to represent milk components or hydrogen peroxide molecules. The completed sensor stack remains visible underneath:

- Borosilicate prism
- Al
- Al_2O_3
- WS_2
- Milk sample
- Use large curved and straight red arrows to indicate the fabrication sequence.
- Style
- Professional journal graphical abstract
- Clean vector illustration
- Soft gradients
- Minimalistic design
- Realistic material colors
- Crisp edges
- Scientific publication quality
- High resolution (4K)
- White background
- Symmetrical composition
- No unnecessary decorative elements
- Clear material labels using a sans-serif font
- Suitable for Nature, ACS, Elsevier, or MDPI journals.
- Color palette
- Borosilicate prism: translucent light blue
- Aluminum: metallic silver-gray

- Aluminum oxide: warm orange-brown
- WS_2: dark slate blue
- Arrows: coral red
- Sample container: transparent glass
- Liquid: pale cream-white
- Water molecules: red oxygen, white hydrogen
- Soft shadows for depth
- Rendering
- Ultra-clean scientific vector illustration, publication-ready, high-resolution infographic, 3D isometric perspective, realistic materials, subtle lighting, sharp text labels, modern academic graphical abstract.
